# Molecular Mimicry between Meningococcal B Factor H-Binding Protein and Human Proteins

**DOI:** 10.1055/s-0043-1776985

**Published:** 2023-11-16

**Authors:** Darja Kanduc

**Affiliations:** 1 Department of Biosciences, Biotechnologies and Biopharmaceutics, University of Bari, Bari, Italy

**Keywords:** molecular mimicry, Trumenba vaccine, factor H-binding protein, human proteome, adverse events

## Abstract

This study calls attention on molecular mimicry and the consequent autoimmune cross reactivity as the molecular mechanism that can cause adverse events following meningococcal B vaccination and warns against active immunizations based on entire antigen.

## Introduction

The bacterium *Neisseria meningitides* (aka meningococcus) can cause a multitude of severe illnesses, collectively termed meningococcal disease. 1 Numerous vaccines are available and, among them, a meningococcal B vaccine (namely, Trumenba) has been approved for children and contains the lipoprotein factor H-binding protein (fHbp). 2 Indeed, as described by Seib et al, 3 preclinical studies demonstrated that fHbp elicits a robust bactericidal antibody response that correlates with the amount of fHbp expressed on the bacterial surface. However, according to the vaccine-prescribing informations, 4 numerous adverse events occur following fHbp vaccine administration. That is, verbatim:

- Immune system disorders.
- Hypersensitivity reactions, including anaphylactic reactions.
- Nervous system disorders.
- Syncope.

Currently, at the best of the author's knowledge, no investigation/hypothesis has been proposed to define the molecular mechanism that triggers the adverse events following the Trumenba vaccine administration. In analyzing the issue, this study posed the question of whether molecular mimicry between the vaccine antigen and the human proteins might play a role as already found in analogous research models. 5 6

## Materials and Methods

Molecular mimicry between the bacterial fHbp antigen and human proteins was analyzed according to published methodology. 5 6 In brief, the fHbp antigen, 274 amino acids (aa) as described at https://www.uniprot.org/uniprotkb/Q9JXV4/ , was dissected into pentapeptides offset each other by one aa residue (i.e., MNRTA, NRTAF, RTAFC, and so forth) and the resulting bacterial pentapeptides were analyzed for occurrences within the human proteome.

Pentapeptides were used since a peptide grouping composed of 5 aa defines a minimal immune unit that can (1) induce highly specific antibodies and (2) determine antigen–antibody specific interaction. 7 8 9 10 Peptide match and peptide search programs available at www.uniprot.org 11 were used.

## Results

Following molecular mimicry analyses, it was found that fHbp pentapeptides repeatedly occur for a total of 2,809 multiple occurrences in human protein alterations which can lead to severe diseases. The bacterial versus human pentapeptide overlap is of such a dimension that obviously it cannot be reported in the context of an article. Consequently, data are reported as Supplementary Table S1 that is a fundamental part of this report.

**Table 1:** Peptides shared between fHbp protein and human proteins that—when altered—are associated with myelination, neuropathy, myopathy, ataxia, etc.

Peptides a	Human proteins: name and associated diseases b c
KNEKL	*Chondroitin sulfate N-acetylgalactosaminyltransferase 1* Neuropathy 12
IGAVL	*Claudin-11. Oligodendrocyte-specific protein.* Autoantigen of autoimmune-demyelinating disease 13
SPELN	*Complex I intermediate-associated protein 30, mitochondrial* Deficiency may be associated with leukodystrophy 14
HAVIS, VDGQL	*Contactin-associated protein 1* Alterations associated with hypomyelinating neuropathy 15
GSDDA	*Delta-1-pyrroline-5-carboxylate synthase* Spastic paraplegia; progressive weakness, and spasticity of the lower limbs. Bladder incontinence, gait difficulties, neuropathy 16
AAKQG	*DNA polymerase subunit gamma-1* Neuronal loss, spongiform degeneration, demyelination 17
KLKND, LADAL	*DnaJ homolog subfamily C member 3* Neurodegeneration, ataxia, peripheral neuropathy, hearing loss 18
SAEVE, VQDSE	*Dystonin* Hypotonia, respiratory, and feeding difficulties 19
LTALQ	*E3 ubiquitin-protein ligase LRSAM1* Disorder of the peripheral nervous system 20
LKLAA, RIGDI	*FYVE* , *RhoGEF* , *and PH domain-containing protein 4* Peripheral nerve demyelination 21
LPEGG	*Gelsolin* Required for normal myelin wrapping 22
GSDDA	*Guanine nucleotide-binding protein subunit beta-4* Disorder of the peripheral nervous system 23
TGKLK	*Laminin subunit alpha-2* Muscular dystrophy 24
KSPEL	*Lysophosphatidylserine lipase ABHD12* Hearing loss, ataxia, retinitis pigmentosa, early-onset cataract 25
GKMVA	*Myelin P2 protein* Neuropathy with progressive weakness and atrophy 26
PEGGR	*Myelin protein P0* Dysmyelinating neuropathy 27
VSRFD	*Neurofibromin* Neuropathy with progressive weakness and atrophy 28
KLPEG	*Patatin-like phospholipase domain-containing protein 6* Mental retardation, spastic paraplegia, ataxia, blindness 29
AEKGS	*Potassium voltage-gated channel subfamily D member 3* S pinocerebellar ataxia, incoordination of gait 30
LADAL	*Prelamin-A/C* Disorder of the peripheral nervous system 31
SGEFQ	*Probable helicase senataxin* Spinocerebellar ataxia, neuropathy, dexterity difficulties 32
SGGGG	*Ribose-5-phosphate isomerize* Leukoencephalopathy, psychomotor retardation 33
NTGKL	*RNA polymerase II subunit A C-terminal domain phosphatase* Cataracts, facial dysmorphism, neuropathy 34
DFAAK GGVAA SAEVE	*Sacsin* Cerebellar ataxia, hypermyelination, mitral valve prolapse 35
SDDAS TGKLK	*Serine/threonine-protein kinase DCLK2* Disorders of neuronal structure 36
AAKQG KSLQS	*Voltage-gated sodium channel subunit alpha Nav1.8* Detrimental to motor axons 37
TSFDK	*Sodium/potassium-transporting ATPase subunit alpha-3* Related to neurological disorders, epilepsy 37
LQSLT, QGAEK	*Solute carrier family 12 member 6* Sensorimotor neuropathy, mental retardation 38
SSGGGG	*Solute carrier family 25 member 46* Peripheral sensorimotor neuropathy 39
KMVAK	*Thioredoxin, mitochondrial* Severe cerebellar atrophy, epilepsy, dystonia, optic atrophy 40
LAAQG	*Thymidine phosphorylase* Leukoencephalopathy, cachexia, neuropathy, myopathy 41
DIGAV	*Trifunctional enzyme subunit alpha, mitochondrial* Hypoglycemia, cardiomyopathy, axonopathy, weakness, hepatic dysfunction, respiratory failure 42
LAAKQG	*Wolframin* Diabetes, sensorineural deafness, dementia 43
SSGGGG	*Zinc finger SWIM domain-containing protein 6* Abnormal gait, autistic features 44
SSGGG	*Beta-1,4 N-acetylgalactosaminyltransferase 1* Axonal degeneration 45

Abbreviation: fHbp, factor H-binding protein.

a Hexapeptides formed by overlapping pentapeptides have been underlined.

b Human protein names are given in italic.

c Further disease details are available in OMIM, PubMed, and UniProt databases.

In parallel, the severe diseases 12 13 14 15 16 17 18 19 20 21 22 23 24 25 26 27 28 29 30 31 32 33 34 35 36 37 38 39 40 41 42 43 44 45 that could derive from the massive molecular mimicry and consequent cross-reactivity and autoimmunity are numerous. Here, only a short synopsis that gives an overview of such diseases is given in Table 1 , thus confirming the vaccine-induced injuries listed in the Trumenba vaccine-prescribing informations. 4

## Conclusion

The vast sharing of immune peptide determinants between the bacterial fHbp antigen and the human proteins warns against Trumenba utilization to prevent meningococcal diseases. Indeed, the present data speak for themselves and clearly predict a very high incidence of autoimmune pathologies in the vaccinees as a result of molecular mimicry and the consequent potential cross-reactivity, thus factually confirming what had already been stated in the prescribing information of the Trumenba vaccine. 4

In synthesis, this report adds to numerous previous studies, 5 6 7 8 9 10 and further references therein, that explicated how only vaccinal protocols based on peptide sequences uniquely present in the pathogenic antigens can provide therapeutic vaccines free of adverse events.
