# Supplementary material for: Molecular Mimicry between Meningococcal B Factor H-Binding Protein and Human Proteins
Source: Glob Med Genet. 2023 Nov 16;10(4):311–4. doi: 10.1055/s-0043-1776985 (PMC10653992; doi:10.1055/s-0043-1776985)
Supplement: Supplementary file 1 — Supplementary Material [file 10-1055-s-0043-1776985-s2300066.pdf]

Supplementary Table S1 List of the 2,809 pentapeptide matches occurring between fHBP antigen and the human proteome (including multiple occurrences)

1.	CSSGG	Coiled-coil domain-containing protein 187
2.	CSSGG	Rho GTPase-activating protein 6
3.	CSSGG	Thymocyte selection-associated high mobility group box protein TOX
4.	CSSGG	Keratin, type II cytoskeletal 4
5.	CSSGG	Receptor tyrosine-protein kinase erbB-3 precursor
6.	CSSGG	Sodium- and chloride-dependent GABA transporter 3
7.	CSSGG	Bifunctional heparan sulfate N-deacetylase/N-sulfotransferase 2
8.	CSSGG	Putative endogenous retrovirus group K member 11-1 Env polyprotein
9.	CSSGG	FH1/FH2 domain-containing protein 3
10.	CSSGG	RNA-binding protein 48
11.	CSSGG	Late cornified envelope protein 1C
12.	CSSGG	Late cornified envelope protein 1D
13.	CSSGG	Late cornified envelope protein 1E
14.	CSSGG	Late cornified envelope protein 1F
15.	CSSGG	Late cornified envelope protein 1A
16.	CSSGG	Late cornified envelope protein 1B
17.	CSSGG	Late cornified envelope protein 2A
18.	CSSGG	Late cornified envelope protein 2D
19.	CSSGG	Adhesion G-protein coupled receptor D1 precursor
20.	CSSGG	Serine dehydratase-like
21.	CSSGG	TRIO and F-actin-binding protein
22.	CSSGG	Tripartite motif-containing protein 45
23.	CSSGG	Hsp70-binding protein 1
24.	CSSGG	Protein inturned
25.	CSSGG	ALK tyrosine kinase receptor precursor
26.	SSGGG	E3 ubiquitin-protein ligase MARCHF11
27.	SSGGG	Zinc finger protein 839
28.	SSGGG	Armadillo repeat protein deleted in velocardiofacial syndrome
29.	SSGGG	Butyrophilin subfamily 3 member A3 precursor
30.	SSGGG	AT-rich interactive domain-containing protein 1A
31.	SSGGG	Kinesin-like protein KIF3C
32.	SSGGG	ERC protein 2
33.	SSGGG	Period circadian protein homolog 1
34.	SSGGG	Rho GTPase-activating protein 6
35.	SSGGG	Zinc finger C3H1 domain-containing protein
36.	SSGGG	Leucine zipper putative tumor suppressor 3
37.	SSGGG	PH domain leucine-rich repeat-containing protein phosphatase 1
38.	SSGGG	Transmembrane and coiled-coil domains protein 2
39.	SSGGG	Nuclear receptor corepressor 1
40.	SSGGG	RNA-binding motif protein, X-linked-like-2
41.	SSGGG	Homeobox protein SIX3
42.	SSGGG	Zinc finger protein ZIC 2
43.	SSGGG	Eomesodermin homolog
44.	SSGGG	Transcription factor AP-1

(*Continued*)

 © 2023. The Author(s).

Supplementary Table S1 (*Continued*)

45.	SSGGG	Keratin, type II cytoskeletal 7
46.	SSGGG	Keratin, type I cytoskeletal 10
47.	SSGGG	Fos-related antigen 2
48.	SSGGG	Synapsin-1
49.	SSGGG	Early growth response protein 1
50.	SSGGG	Keratin, type I cytoskeletal 15
51.	SSGGG	MHC class II regulatory factor RFX1
52.	SSGGG	Glycine dehydrogenase (decarboxylating), mitochondrial precursor
53.	SSGGG	Loricrin
54.	SSGGG	Collagen alpha-2 (VIII) chain precursor
55.	SSGGG	Transcriptional repressor protein YY1
56.	SSGGG	Homeobox protein Hox-D11
57.	SSGGG	Keratin, type I cytoskeletal 9
58.	SSGGG	RNA-binding protein FUS
59.	SSGGG	Nuclear pore complex protein Nup214
60.	SSGGG	Keratin, type II cytoskeletal 2 epidermal
61.	SSGGG	Transcription factor GATA-4
62.	SSGGG	Protein PRRC2A
63.	SSGGG	Glycogen synthase kinase-3 alpha
64.	SSGGG	Carnitine O-palmitoyltransferase 1, liver isoform
65.	SSGGG	Growth arrest-specific protein 1 precursor
66.	SSGGG	Protein AF-17
67.	SSGGG	Protein ripply3
68.	SSGGG	Ras-related protein Rab-10
69.	SSGGG	Putative endogenous retrovirus group K member 11-1 Env polyprotein
70.	SSGGG	Eukaryotic translation initiation factor 4 gamma 2
71.	SSGGG	Homeobox protein Nkx-6.1
72.	SSGGG	Beta-1,4 N-acetylgalactosaminyltransferase 1
73.	SSGGG	Potassium voltage-gated channel subfamily C member 4
74.	SSGGG	Transcriptional activator MN1
75.	SSGGG	Calcium-activated potassium channel subunit alpha-1
76.	SSGGG	POU domain, class 4, transcription factor 2
77.	SSGGG	Forkhead box protein C1
78.	SSGGG	RE1-silencing transcription factor
79.	SSGGG	Cyclin-dependent kinase 13
80.	SSGGG	H2.0-like homeobox protein
81.	SSGGG	NK1 transcription factor-related protein 1
82.	SSGGG	Corneodesmosin precursor
83.	SSGGG	Twist-related protein 1
84.	SSGGG	Forkhead box protein D1
85.	SSGGG	AP2-associated protein kinase 1
86.	SSGGG	E3 ubiquitin-protein ligase TRIM71
87.	SSGGG	Golgin subfamily A member 7B
88.	SSGGG	FH1/FH2 domain-containing protein 3

 © 2023. The Author(s).

Supplementary Table S1 (*Continued*)

89.	SSGGG	Midnolin
90.	SSGGG	Ankyrin repeat domain-containing protein SOWAHC
91.	SSGGG	Late cornified envelope protein 1C
92.	SSGGG	Late cornified envelope protein 1D
93.	SSGGG	Late cornified envelope protein 1E
94.	SSGGG	Late cornified envelope protein 1F
95.	SSGGG	Late cornified envelope protein 1A
96.	SSGGG	Late cornified envelope protein 1B
97.	SSGGG	Late cornified envelope protein 2A
98.	SSGGG	Late cornified envelope protein 2D
99.	SSGGG	Rho GTPase-activating protein 17
100.	SSGGG	Nipped-B-like protein
101.	SSGGG	INO80 complex subunit C
102.	SSGGG	Monofunctional C1-tetrahydrofolate synthase, mitochondrial precursor
103.	SSGGG	Ran-binding protein 10
104.	SSGGG	RNA-binding protein MEX3B
105.	SSGGG	Zinc finger protein AEBP2
106.	SSGGG	Inhibitor of nuclear factor kappa-B kinase-interacting protein
107.	SSGGG	Putative serine protease 42 precursor
108.	SSGGG	Mucin-19 precursor
109.	SSGGG	TBC1 domain family member 1
110.	SSGGG	LIM domain-binding protein 1
111.	SSGGG	Gametogenetin
112.	SSGGG	Ankyrin repeat and KH domain-containing protein 1
113.	SSGGG	C-Maf-inducing protein
114.	SSGGG	Elongin-A2
115.	SSGGG	Transcription initiation factor TFIID subunit 1-like
116.	SSGGG	Usher syndrome type-1C protein-binding protein 1
117.	SSGGG	Neuron navigator 1
118.	SSGGG	Class E basic helix–loop–helix protein 22
119.	SSGGG	Zinc transporter 5
120.	SSGGG	Spermatogenesis-associated protein 20 precursor
121.	SSGGG	Carnitine O-palmitoyltransferase 1, brain isoform
122.	SSGGG	E3 ubiquitin-protein ligase DTX3L
123.	SSGGG	Dual specificity protein phosphatase 19
124.	SSGGG	Zinc finger CCHC domain-containing protein 14
125.	SSGGG	TATA-binding protein-associated factor 2N
126.	SSGGG	Transcription factor IIIB 90 kDa subunit
127.	SSGGG	Solute carrier family 25 member 46
128.	SSGGG	Solute carrier family 41 member 2
129.	SSGGG	Zinc finger CCCH domain-containing protein 10
130.	SSGGG	Rho guanine nucleotide exchange factor 17
131.	SSGGG	Serine/threonine-protein kinase SMG1
132.	SSGGG	RNA-binding protein 15

(*Continued*)

 © 2023. The Author(s).

Supplementary Table S1 (*Continued*)

133.	SSGGG	Docking protein 1
134.	SSGGG	Cbp/p300-interacting transactivator 2
135.	SSGGG	Membrane-associated phosphatidylinositol transfer protein 2
136.	SSGGG	ATP-dependent DNA/RNA helicase DHX36
137.	SSGGG	Zinc finger and BTB domain-containing protein 3
138.	SSGGG	Cadherin-related family member 5 precursor
139.	SSGGG	Chromobox protein homolog 8
140.	SSGGG	Zinc finger SWIM domain-containing protein 6
141.	SSGGG	WD repeat-containing protein 6
142.	SSGGG	Serine protease inhibitor Kazal-type 5 precursor
143.	SSGGG	Delta-like protein 4 precursor
144.	SSGGG	Ubiquilin-4
145.	SSGGG	CREB/ATF bZIP transcription factor
146.	SSGGG	Sal-like protein 1
147.	SSGGG	Cyclin-dependent kinase 12
148.	SSGGG	Hsp70-binding protein 1
149.	SSGGG	Zinc finger and BTB domain-containing protein 4
150.	SSGGG	Protein RCC2
151.	SSGGG	Ena/VASP-like protein
152.	SSGGG	Bromodomain adjacent to zinc finger domain protein 2B
153.	SSGGG	Solute carrier organic anion transporter family member 3A1
154.	SSGGG	Calcium-dependent secretion activator 1
155.	SSGGG	ALK tyrosine kinase receptor precursor
156.	SSGGG	Collagen alpha-1(XVII) chain
157.	SSGGG	Hermansky-Pudlak syndrome 5 protein
158.	SSGGG	AFG3-like protein 2 precursor
159.	SSGGG	Nuclear receptor corepressor 2
160.	SSGGG	TAF6-like RNA polymerase II p300/CBP-associated factor-associated factor 65 kDa subunit 6L
161.	SSGGG	Calmodulin-binding transcription activator 1
162.	SGGGG	Homeobox protein unc-4 homolog
163.	SGGGG	Coiled-coil domain-containing protein 85C
164.	SGGGG	Fidgetin-like protein 2
165.	SGGGG	Proline-rich protein 33
166.	SGGGG	IQCJ-SCHIP1 readthrough transcript protein
167.	SGGGG	C2 calcium-dependent domain-containing protein 4D
168.	SGGGG	Uncharacterized protein C4orf54
169.	SGGGG	Uncharacterized membrane protein C3orf80 precursor
170.	SGGGG	Dynamin-1-like protein
171.	SGGGG	ATP-dependent RNA helicase DDX3X
172.	SGGGG	Transcription factor E2F3
173.	SGGGG	AT-rich interactive domain-containing protein 1A
174.	SGGGG	Tumor necrosis factor receptor superfamily member 10B precursor
175.	SGGGG	Kinesin-like protein KIF3C
176.	SGGGG	Kinesin-like protein KIF3B

 © 2023. The Author(s).

Supplementary Table S1 (*Continued*)

177.	SGGGG	ERC protein 2
178.	SGGGG	Ephrin type-B receptor 6 precursor
179.	SGGGG	ATP-dependent RNA helicase DDX3Y
180.	SGGGG	Mothers against decapentaplegic homolog 6
181.	SGGGG	A-kinase anchor protein 8
182.	SGGGG	Zinc finger C3H1 domain-containing protein
183.	SGGGG	DnaJ homolog subfamily A member 2 precursor
184.	SGGGG	CCR4-NOT transcription complex subunit 3
185.	SGGGG	Ankyrin repeat domain-containing protein 17
186.	SGGGG	DnaJ homolog subfamily C member 8
187.	SGGGG	Triple functional domain protein
188.	SGGGG	SLIT and NTRK-like protein 3 precursor
189.	SGGGG	Zinc finger protein ZIC 2
190.	SGGGG	Lymphocyte antigen 86 precursor
191.	SGGGG	Eomesodermin homolog
192.	SGGGG	One cut domain family member 2
193.	SGGGG	Keratin, type II cytoskeletal 1
194.	SGGGG	Homeobox protein Hox-C6
195.	SGGGG	Schwannomin-interacting protein 1
196.	SGGGG	Zinc finger protein GLI2
197.	SGGGG	Keratin, type I cytoskeletal 10
198.	SGGGG	Keratin, type II cytoskeletal 5
199.	SGGGG	Homeobox protein Hox-B3
200.	SGGGG	Serine/threonine-protein kinase B-raf
201.	SGGGG	Fos-related antigen 2
202.	SGGGG	Desmoplakin
203.	SGGGG	Synapsin-1
204.	SGGGG	Potassium voltage-gated channel subfamily A member 6
205.	SGGGG	Insulin-like growth factor-binding protein 2 precursor
206.	SGGGG	Early growth response protein 1
207.	SGGGG	Keratin, type I cytoskeletal 15
208.	SGGGG	Homeobox protein engrailed-2
209.	SGGGG	Nuclear factor NF-kappa-B p105 subunit
210.	SGGGG	Cartilage matrix protein precursor
211.	SGGGG	MHC class II regulatory factor RFX1
212.	SGGGG	Loricrin
213.	SGGGG	Transcriptional repressor protein YY1
214.	SGGGG	Ephrin type-A receptor 8 precursor
215.	SGGGG	SHC-transforming protein 1
216.	SGGGG	Homeobox protein Hox-A10
217.	SGGGG	Homeobox protein Hox-A11
218.	SGGGG	Serpin B6
219.	SGGGG	Keratin, type I cytoskeletal 9
220.	SGGGG	RNA-binding protein FUS

(*Continued*)

Supplementary Table S1 (*Continued*)

221.	SGGGG	Transcription factor SOX-11
222.	SGGGG	Keratin, type II cytoskeletal 2 epidermal
223.	SGGGG	Transcription factor 7
224.	SGGGG	Adenylate cyclase type 8
225.	SGGGG	Transcription factor SOX-3
226.	SGGGG	Homeobox protein Nkx-2.1
227.	SGGGG	Dual specificity mitogen-activated protein kinase kinase 4
228.	SGGGG	Rap1 GTPase-activating protein 1
229.	SGGGG	mRNA decay activator protein ZFP36L2
230.	SGGGG	Transcription factor SOX-2
231.	SGGGG	Protein PRRC2A
232.	SGGGG	Ribose-5-phosphate isomerase
233.	SGGGG	Homeobox even-skipped homolog protein 1
234.	SGGGG	Glycogen synthase kinase-3 alpha
235.	SGGGG	ETS domain-containing transcription factor ERF
236.	SGGGG	Vasodilator-stimulated phosphoprotein
237.	SGGGG	Heat shock-related 70 kDa protein 2
238.	SGGGG	Growth arrest-specific protein 1 precursor
239.	SGGGG	Solute carrier family 12 member 2
240.	SGGGG	Protein AF-17
241.	SGGGG	Eukaryotic translation initiation factor 4 gamma 2
242.	SGGGG	Homeobox protein Nkx-6.1
243.	SGGGG	SKI family transcriptional corepressor 1
244.	SGGGG	Zinc finger X-linked protein ZXDB
245.	SGGGG	Cyclin-dependent kinase 16
246.	SGGGG	Transcriptional activator protein Pur-alpha
247.	SGGGG	Keratin, type II cytoskeletal 2 oral
248.	SGGGG	Contactin-2 precursor
249.	SGGGG	Transcription factor Sp3
250.	SGGGG	Histone-lysine N-methyltransferase 2A
251.	SGGGG	Potassium voltage-gated channel subfamily C member 4
252.	SGGGG	Transcription factor SOX-4
253.	SGGGG	Transcriptional activator MN1
254.	SGGGG	Calcium-activated potassium channel subunit alpha-1
255.	SGGGG	POU domain, class 4, transcription factor 2
256.	SGGGG	Interleukin enhancer-binding factor 3
257.	SGGGG	Forkhead box protein C1
258.	SGGGG	RE1-silencing transcription factor
259.	SGGGG	Translation initiation factor eIF-2B subunit epsilon
260.	SGGGG	Heterogeneous nuclear ribonucleoprotein A0
261.	SGGGG	Serine/arginine-rich splicing factor 6
262.	SGGGG	Transformer-2 protein homolog alpha
263.	SGGGG	Sorting nexin-1
264.	SGGGG	Pumilio homolog 1

 © 2023. The Author(s).

Supplementary Table S1 (*Continued*)

265.	SGGGG	H2.0-like homeobox protein
266.	SGGGG	Polycomb protein SUZ12
267.	SGGGG	NK1 transcription factor-related protein 1
268.	SGGGG	Nuclear receptor subfamily 6 group A member 1
269.	SGGGG	Homeobox protein TGIF1
270.	SGGGG	Urea transporter 2
271.	SGGGG	Zinc finger homeobox protein 3
272.	SGGGG	Coiled-coil domain-containing protein 6
273.	SGGGG	Mitogen-activated protein kinase kinase kinase 11
274.	SGGGG	Transmembrane protein 132A precursor
275.	SGGGG	AP2-associated protein kinase 1
276.	SGGGG	E3 ubiquitin-protein ligase TRIM71
277.	SGGGG	Tumor suppressor candidate gene 1 protein
278.	SGGGG	Golgin subfamily A member 7B
279.	SGGGG	Midnolin
280.	SGGGG	Fibronectin type III domain-containing protein 3B
281.	SGGGG	DBIRD complex subunit ZNF326
282.	SGGGG	Potassium channel subfamily T member 1
283.	SGGGG	Protein phosphatase 1 regulatory subunit 29 precursor
284.	SGGGG	NHS-like protein 1
285.	SGGGG	Solute carrier family 35 member F1
286.	SGGGG	OTU domain-containing protein 3
287.	SGGGG	Late cornified envelope protein 1C
288.	SGGGG	Late cornified envelope protein 1D
289.	SGGGG	Late cornified envelope protein 1E
290.	SGGGG	Late cornified envelope protein 1F
291.	SGGGG	Late cornified envelope protein 1A
292.	SGGGG	Late cornified envelope protein 1B
293.	SGGGG	Late cornified envelope protein 2A
294.	SGGGG	Late cornified envelope protein 2D
295.	SGGGG	Protein SOGA3 precursor
296.	SGGGG	Leucine-rich repeat and calponin homology domain-containing protein 2
297.	SGGGG	Doublesex- and mab-3-related transcription factor A1
298.	SGGGG	Terminal nucleotidyltransferase 4A
299.	SGGGG	DDB1- and CUL4-associated factor 15
300.	SGGGG	Kazrin
301.	SGGGG	Nuclear apoptosis-inducing factor 1
302.	SGGGG	DENN domain-containing protein 5A
303.	SGGGG	Zinc finger protein 746
304.	SGGGG	Putative ATP-dependent RNA helicase DHX57
305.	SGGGG	Protein FAM117B
306.	SGGGG	F-box only protein 46
307.	SGGGG	Monofunctional C1-tetrahydrofolate synthase, mitochondrial precursor
308.	SGGGG	Transmembrane protein 64

(*Continued*)

 © 2023. The Author(s).

Supplementary Table S1 (*Continued*)

309.	SGGGG	E3 ubiquitin-protein ligase RNF19B
310.	SGGGG	RNA-binding protein MEX3B
311.	SGGGG	Zinc finger protein AEBP2
312.	SGGGG	Tripartite motif-containing protein 67
313.	SGGGG	Putative uncharacterized protein ZNF516-DT
314.	SGGGG	Ubiquitin carboxyl-terminal hydrolase 51
315.	SGGGG	Inhibitor of nuclear factor kappa-B kinase-interacting protein
316.	SGGGG	7SK snRNA methylphosphate capping enzyme
317.	SGGGG	Lysine-specific demethylase 3B
318.	SGGGG	Pogo transposable element with ZNF domain
319.	SGGGG	Retinoic acid-induced protein 1
320.	SGGGG	Mucin-19 precursor
321.	SGGGG	Cytoplasmic polyadenylation element-binding protein 2
322.	SGGGG	TBC1 domain family member 1
323.	SGGGG	Gametogenetin
324.	SGGGG	Leucine-rich adaptor protein 1-like
325.	SGGGG	Contactin-4 precursor
326.	SGGGG	E3 ubiquitin-protein ligase UBR2
327.	SGGGG	Ankyrin repeat and KH domain-containing protein 1
328.	SGGGG	Transcription factor Sp8
329.	SGGGG	C-Maf-inducing protein
330.	SGGGG	Biorientation of chromosomes in cell division protein 1-like 2
331.	SGGGG	Integral membrane protein GPR137C
332.	SGGGG	MPN domain-containing protein
333.	SGGGG	POU domain, class 5, transcription factor 2
334.	SGGGG	APC membrane recruitment protein 2
335.	SGGGG	Reticulophagy regulator 2
336.	SGGGG	Protein FAM98A
337.	SGGGG	Putative RNA-binding protein 15B
338.	SGGGG	Phospholipase DDHD1
339.	SGGGG	AT-rich interactive domain-containing protein 1B
340.	SGGGG	Class E basic helix–loop–helix protein 22
341.	SGGGG	Neurobeachin
342.	SGGGG	Collagen alpha-1 (XXII) chain precursor
343.	SGGGG	Probable E3 ubiquitin-protein ligase RNF217
344.	SGGGG	Chromodomain-helicase-DNA-binding protein 6
345.	SGGGG	Transient receptor potential cation channel subfamily M member 4
346.	SGGGG	Serine/threonine-protein kinase BRSK1
347.	SGGGG	Ankyrin repeat domain-containing protein 24
348.	SGGGG	GATA zinc finger domain-containing protein 1
349.	SGGGG	Intelectin-1 precursor
350.	SGGGG	Intelectin-2 precursor
351.	SGGGG	Zinc finger CCHC domain-containing protein 14
352.	SGGGG	SHC-transforming protein 3

 © 2023. The Author(s).

Supplementary Table S1 (*Continued*)

353.	SGGGG	Ankyrin repeat and SAM domain-containing protein 1A
354.	SGGGG	Keratin, type I cuticular Ha5
355.	SGGGG	Solute carrier family 25 member 46
356.	SGGGG	Zinc finger and BTB domain-containing protein 9
357.	SGGGG	Protein FAM122A
358.	SGGGG	E3 ubiquitin-protein ligase CHFR
359.	SGGGG	RNA-binding protein 33
360.	SGGGG	Zinc finger protein 503
361.	SGGGG	Biorientation of chromosomes in cell division protein 1
362.	SGGGG	Zinc finger protein 469
363.	SGGGG	Protocadherin-16 precursor
364.	SGGGG	Protein arginine N-methyltransferase 6
365.	SGGGG	Ras/Rap GTPase-activating protein SynGAP
366.	SGGGG	Serine/threonine-protein kinase SMG1
367.	SGGGG	Membrane-associated guanylate kinase, WW and PDZ domain-containing protein 1
368.	SGGGG	Zinc finger protein ZIC 5
369.	SGGGG	Protein Niban 2
370.	SGGGG	Heterogeneous nuclear ribonucleoprotein A/B
371.	SGGGG	Heterogeneous nuclear ribonucleoprotein U-like protein 1
372.	SGGGG	Oxysterol-binding protein-related protein 10
373.	SGGGG	Membrane-associated phosphatidylinositol transfer protein 2
374.	SGGGG	Zinc finger protein GLIS2
375.	SGGGG	Reticulon-4 receptor precursor
376.	SGGGG	ATP-dependent DNA/RNA helicase DHX36
377.	SGGGG	Transmembrane protein 245
378.	SGGGG	Calsyntenin-2 precursor
379.	SGGGG	PR domain zinc finger protein 13
380.	SGGGG	Pecanex-like protein 3
381.	SGGGG	Zinc finger protein 703
382.	SGGGG	Leucine-rich repeat-containing G-protein coupled receptor 6 precursor
383.	SGGGG	Anoctamin-8
384.	SGGGG	Zinc finger SWIM domain-containing protein 6
385.	SGGGG	Transcription factor 7-like 1
386.	SGGGG	Serine protease inhibitor Kazal-type 5 precursor
387.	SGGGG	PR domain zinc finger protein 8
388.	SGGGG	SH2B adapter protein 1
389.	SGGGG	Sal-like protein 1
390.	SGGGG	Leucine-rich repeat-containing protein 4B precursor
391.	SGGGG	Zinc finger CCHC domain-containing protein 3
392.	SGGGG	Cerebral cavernous malformations 2 protein-like
393.	SGGGG	CUE domain-containing protein 1
394.	SGGGG	Protein FAM53C
395.	SGGGG	Hsp70-binding protein 1
396.	SGGGG	Zinc finger and BTB domain-containing protein 4

(*Continued*)

 © 2023. The Author(s).

Supplementary Table S1 (*Continued*)

397.	SGGGG	Protocadherin-10 precursor
398.	SGGGG	Methyl-CpG-binding domain protein 2
399.	SGGGG	Tumor necrosis factor receptor superfamily member 10D precursor
400.	SGGGG	Gamma-aminobutyric acid type B receptor subunit 1 precursor
401.	SGGGG	Gamma-adducin
402.	SGGGG	Signal recognition particle subunit SRP68
403.	SGGGG	Large neutral amino acids transporter small subunit 2
404.	SGGGG	Ena/VASP-like protein
405.	SGGGG	Dachshund homolog 1
406.	SGGGG	Ventral anterior homeobox 2
407.	SGGGG	tRNA (adenine(58)-N(1))-methyltransferase non-catalytic subunit TRM6
408.	SGGGG	Lymphoid enhancer-binding factor 1
409.	SGGGG	Protein TASOR
410.	SGGGG	Peroxisomal carnitine O-octanoyltransferase
411.	SGGGG	RNA-binding protein Raly
412.	SGGGG	Zinc finger protein 777
413.	SGGGG	YEATS domain-containing protein 2
414.	SGGGG	ALK tyrosine kinase receptor precursor
415.	SGGGG	Collagen alpha-1 (XVII) chain
416.	SGGGG	Synergin gamma
417.	SGGGG	Protein bassoon
418.	SGGGG	E3 ubiquitin-protein ligase TRIM33
419.	SGGGG	Dexamethasone-induced Ras-related protein 1 precursor
420.	SGGGG	Zinc finger protein 281
421.	SGGGG	AFG3-like protein 2 precursor
422.	SGGGG	AMME syndrome candidate gene 1 protein
423.	SGGGG	SH3 and multiple ankyrin repeat domains protein 1
424.	SGGGG	Nuclear receptor corepressor 2
425.	SGGGG	Heparan sulfate glucosamine 3-O-sulfotransferase 4
426.	SGGGG	TAF6-like RNA polymerase II p300/CBP-associated factor-associated factor 65 kDa subunit 6L
427.	GGGGV	ADP-ribose glycohydrolase MACROD2
428.	GGGGV	Pecanex-like protein 2
429.	GGGGV	Cilia- and flagella-associated protein 99
430.	GGGGV	Dynamin-1-like protein
431.	GGGGV	Segment polarity protein dishevelled homolog DVL-2
432.	GGGGV	Short stature homeobox protein 2
433.	GGGGV	F-box only protein 24
434.	GGGGV	Usherin precursor
435.	GGGGV	Ras-related protein R-Ras precursor
436.	GGGGV	Keratin, type I cytoskeletal 10
437.	GGGGV	Atrial natriuretic peptide receptor 3 precursor
438.	GGGGV	Alpha-1D adrenergic receptor
439.	GGGGV	Neuromedin-K receptor
440.	GGGGV	Serine/threonine-protein kinase 19

 © 2023. The Author(s).

Supplementary Table S1 (*Continued*)

441.	GGGGV	Pancreas/duodenum homeobox protein 1
442.	GGGGV	FXYD domain-containing ion transport regulator 7
443.	GGGGV	BPI fold-containing family B member 4 precursor
444.	GGGGV	Ras-related protein R-Ras2 precursor
445.	GGGGV	Desmocollin-2 precursor
446.	GGGGV	Dual specificity protein phosphatase 5
447.	GGGGV	Tumor suppressor candidate gene 1 protein
448.	GGGGV	Intermediate filament family orphan 2
449.	GGGGV	Arylacetamide deacetylase-like 3
450.	GGGGV	Transmembrane anterior posterior transformation protein 1 homolog
451.	GGGGV	Mitotic deacetylase-associated SANT domain protein
452.	GGGGV	Transmembrane protein 64
453.	GGGGV	Serine/threonine-protein kinase LMTK1
454.	GGGGV	Zinc finger protein AEBP2
455.	GGGGV	Mucin-19 precursor
456.	GGGGV	B-cell CLL/lymphoma 9-like protein
457.	GGGGV	Glucocorticoid-induced transcript 1 protein
458.	GGGGV	RNA-binding protein MEX3D
459.	GGGGV	EH domain-binding protein 1-like protein 1
460.	GGGGV	Zinc finger protein 276
461.	GGGGV	Plasminogen activator inhibitor 1 RNA-binding protein
462.	GGGGV	Class E basic helix–loop–helix protein 22
463.	GGGGV	Far upstream element-binding protein 1
464.	GGGGV	Endoplasmic reticulum lectin 1 precursor
465.	GGGGV	Zinc finger protein 503
466.	GGGGV	Unconventional myosin-XVB
467.	GGGGV	MAP/microtubule affinity-regulating kinase 4
468.	GGGGV	Proton myo-inositol cotransporter
469.	GGGGV	ADP-ribose glycohydrolase MACROD1
470.	GGGGV	B-cell lymphoma/leukemia 11B
471.	GGGGV	SH2B adapter protein 1
472.	GGGGV	Disrupted in schizophrenia 1 protein
473.	GGGGV	Metal transporter CNNM1
474.	GGGGV	Leucine-rich repeat-containing protein 4B precursor
475.	GGGGV	Protocadherin-10 precursor
476.	GGGGV	Gamma-adducin
477.	GGGGV	Large neutral amino acids transporter small subunit 2
478.	GGGGV	Glutathione hydrolase 7 precursor
479.	GGGGV	Calcium load-activated calcium channel
480.	GGGGV	Collagen alpha-1(XVII) chain
481.	GGGGV	Cytosolic iron-sulfur assembly component 2B
482.	GGGVA	Laforin, isoform 9
483.	GGGVA	Isocitrate dehydrogenase [NADP] cytoplasmic
484.	GGGVA	Interferon alpha-inducible protein 27, mitochondrial precursor

(*Continued*)

 © 2023. The Author(s).

Supplementary Table S1 (*Continued*)

485.	GGGVA	Pancreas/duodenum homeobox protein 1
486.	GGGVA	NAD(P) transhydrogenase, mitochondrial precursor
487.	GGGVA	Protein Tob2
488.	GGGVA	Scaffold attachment factor B2
489.	GGGVA	Dual specificity protein phosphatase 5
490.	GGGVA	Transmembrane protein 132A precursor
491.	GGGVA	Interferon alpha-inducible protein 27-like protein 1
492.	GGGVA	Sal-like protein 3
493.	GGGVA	39S ribosomal protein L4, mitochondrial
494.	GGGVA	Glycosyltransferase 8 domain-containing protein 2
495.	GGGVA	SH2B adapter protein 1
496.	GGGVA	Metal transporter CNNM1
497.	GGGVA	Leucine-rich repeat-containing protein 4B precursor
498.	GGGVA	Large neutral amino acids transporter small subunit 2
499.	GGGVA	Dachshund homolog 1
500.	GGVAA	Laforin, isoform 9
501.	GGVAA	Uncharacterized membrane protein C3orf80 precursor
502.	GGVAA	Lysine-specific demethylase 6B
503.	GGVAA	Tetraspanin-1
504.	GGVAA	Sperm-associated antigen 6
505.	GGVAA	Band 3 anion transport protein
506.	GGVAA	ADP/ATP translocase 2
507.	GGVAA	Lipoprotein lipase precursor
508.	GGVAA	ADP/ATP translocase 1
509.	GGVAA	Elastin precursor
510.	GGVAA	Oxysterol-binding protein 1
511.	GGVAA	Aldehyde dehydrogenase, dimeric NADP-preferring
512.	GGVAA	Glutathione synthetase
513.	GGVAA	NACHT, LRR, and PYD domain-containing protein 5
514.	GGVAA	Dual specificity protein phosphatase 5
515.	GGVAA	Putative uncharacterized protein FLJ44636
516.	GGVAA	PDZ domain-containing protein 4
517.	GGVAA	ATP-dependent RNA helicase DDX1
518.	GGVAA	Interferon alpha-inducible protein 27-like protein 1
519.	GGVAA	Nucleus accumbens-associated protein 1
520.	GGVAA	Msx2-interacting protein
521.	GGVAA	Telomerase protein component 1
522.	GGVAA	Sal-like protein 3
523.	GGVAA	Protein amnionless precursor
524.	GGVAA	ADP/ATP translocase 4
525.	GGVAA	Neurogenin-2
526.	GGVAA	Putative sodium-coupled neutral amino acid transporter 7
527.	GGVAA	Sacsin
528.	GGVAA	Zinc finger SWIM domain-containing protein 5

Supplementary Table S1 (*Continued*)

529.	GGVAA	E3 ubiquitin-protein ligase TRIM33
530.	GGVAA	Low-density lipoprotein receptor-related protein 12 precursor
531.	GVAAD	Proteasome subunit beta type-10 precursor
532.	GVAAD	Malate dehydrogenase, mitochondrial precursor
533.	GVAAD	Semaphorin-3B precursor
534.	GVAAD	Protogenin precursor
535.	GVAAD	Synapse differentiation-inducing gene protein 1
536.	VAADI	DNA polymerase zeta catalytic subunit
537.	VAADI	Signal recognition particle receptor subunit alpha
538.	VAADI	Centrosomal protein kizuna
539.	VAADI	Protein kinase C-binding protein 1
540.	VAADI	Neurogenic locus notch homolog protein 3 precursor
541.	VAADI	Major intrinsically disordered Notch2-binding receptor 1
542.	AADIG	Probable phospholipid-transporting ATPase VB
543.	AADIG	F-box only protein 21
544.	AADIG	Fibrocystin precursor
545.	AADIG	Calcium-transporting ATPase type 2C member 1
546.	AADIG	Unconventional myosin-XVIIIb
547.	AADIG	Titin
548.	ADIGA	Zinc finger protein 646
549.	ADIGA	Nuclear valosin-containing protein-like
550.	ADIGA	Wiskott-Aldrich syndrome protein
551.	ADIGA	MARVEL domain-containing protein 3
552.	ADIGA	N-acetylmuramoyl-L-alanine amidase precursor
553.	ADIGA	PHD and RING finger domain-containing protein 1
554.	ADIGA	Cadherin-7 precursor
555.	ADIGA	Protocadherin alpha-1 precursor
556.	DIGAV	Trifunctional enzyme subunit alpha, mitochondrial precursor
557.	DIGAV	DNA primase small subunit
558.	DIGAV	FRAS1-related extracellular matrix protein 1 precursor
559.	DIGAV	GATOR complex protein WDR59
560.	DIGAV	Isoaspartyl peptidase/L-asparaginase precursor
561.	IGAVL	Cytochrome b5 type B precursor
562.	IGAVL	Adhesion G protein-coupled receptor B2 precursor
563.	IGAVL	Claudin-11
564.	IGAVL	Platelet glycoprotein 4
565.	IGAVL	D(4) dopamine receptor
566.	IGAVL	Aquaporin-4
567.	IGAVL	Glutamate receptor ionotropic, NMDA 1 precursor
568.	IGAVL	Nucleolar GTP-binding protein 2
569.	IGAVL	Myogenesis-regulating glycosidase
570.	IGAVL	RNA polymerase-associated protein CTR9 homolog
571.	IGAVL	Biorientation of chromosomes in cell division protein 1-like 1
572.	IGAVL	Olfactory receptor 1S2

(*Continued*)

Supplementary Table S1 (*Continued*)

573.	IGAVL	Olfactory receptor 6N2
574.	IGAVL	Olfactory receptor 1S1
575.	IGAVL	N6-adenosine-methyltransferase non-catalytic subunit
576.	GAVLA	Sulfhydryl oxidase 1 precursor
577.	GAVLA	Peripheral plasma membrane protein CASK
578.	GAVLA	Mitochondrial import inner membrane translocase subunit Tim17-B
579.	GAVLA	Alpha-1B-glycoprotein precursor
580.	GAVLA	Platelet glycoprotein 4
581.	GAVLA	D(4) dopamine receptor
582.	GAVLA	Cadherin-3 precursor
583.	GAVLA	UDP-glucuronosyltransferase 1A4 precursor
584.	GAVLA	DNA-binding protein RFX5
585.	GAVLA	Sulfite oxidase, mitochondrial precursor
586.	GAVLA	Aquaporin-5
587.	GAVLA	Aquaporin-4
588.	GAVLA	Actin-related protein 2
589.	GAVLA	Mitogen-activated protein kinase kinase kinase 9
590.	GAVLA	Ragulator complex protein LAMTOR4
591.	GAVLA	Hypermethylated in cancer 1 protein
592.	GAVLA	Leucine-rich repeat-containing protein 75B
593.	GAVLA	RNA polymerase-associated protein CTR9 homolog
594.	GAVLA	WD repeat-containing protein 87
595.	GAVLA	Otogelin precursor
596.	GAVLA	Probable G-protein coupled receptor 142
597.	GAVLA	E3 ubiquitin-protein ligase UBR1
598.	GAVLA	Major facilitator superfamily domain-containing protein 9
599.	GAVLA	MARVEL domain-containing protein 3
600.	GAVLA	Multidrug resistance-associated protein 9
601.	GAVLA	Trace amine-associated receptor 6
602.	GAVLA	Trace amine-associated receptor 9
603.	GAVLA	Hemicentin-1 precursor
604.	GAVLA	Tyrosine-protein phosphatase non-receptor type 18
605.	GAVLA	Actin-related protein 8
606.	GAVLA	Transmembrane protein 160
607.	GAVLA	Stabilin-1 precursor
608.	GAVLA	Intraflagellar transport protein 80 homolog
609.	GAVLA	Kinesin-like protein KIF25
610.	GAVLA	Intercellular adhesion molecule 5 precursor
611.	AVLAD	Histone-lysine N-methyltransferase SETD1A
612.	AVLAD	Protein Mis18-beta
613.	AVLAD	Beta-adrenergic receptor kinase 1
614.	AVLAD	Beta-adrenergic receptor kinase 2
615.	AVLAD	Actin-related protein 2
616.	AVLAD	Aldehyde oxidase

 © 2023. The Author(s).

Supplementary Table S1 (*Continued*)

617.	AVLAD	Microfibrillar-associated protein 5 precursor
618.	AVLAD	Activin receptor type-2B precursor
619.	AVLAD	Bifunctional arginine demethylase and lysyl-hydroxylase JMJD6
620.	AVLAD	Membrane progestin receptor delta
621.	AVLAD	Amphoterin-induced protein 3 precursor
622.	AVLAD	Uncharacterized protein C10orf67, mitochondrial precursor
623.	AVLAD	Uncharacterized protein KIAA2013 precursor
624.	AVLAD	TBC1 domain family member 19
625.	AVLAD	tRNA N(3)-methylcytidine methyltransferase METTL2A
626.	AVLAD	Multidrug resistance-associated protein 9
627.	AVLAD	Cyclic AMP-dependent transcription factor ATF-6 beta
628.	AVLAD	Caseinolytic peptidase B protein homolog precursor
629.	AVLAD	Regulator of cell cycle RGCC
630.	AVLAD	Actin-related protein 8
631.	AVLAD	Guanine nucleotide-binding protein-like 3-like protein
632.	VLADA	Kinesin-like protein KIF28P
633.	VLADA	BAI1-associated protein 3
634.	VLADA	Retinal dehydrogenase 1
635.	VLADA	UDP-glucuronosyltransferase 2B4 precursor
636.	VLADA	Phosphatidylinositol 3-kinase regulatory subunit alpha
637.	VLADA	Aldehyde dehydrogenase X, mitochondrial precursor
638.	VLADA	Importin subunit alpha-5
639.	VLADA	40S ribosomal protein S15a
640.	VLADA	Double-stranded RNA-specific editase 1
641.	VLADA	Uncharacterized protein KIAA2013 precursor
642.	VLADA	Olfactory receptor 13A1
643.	VLADA	Roundabout homolog 3 precursor
644.	VLADA	Transcription factor SOX-7
645.	VLADA	Tetratricopeptide repeat protein 12
646.	VLADA	G patch domain-containing protein 2-like
647.	VLADA	DNA helicase MCM9
648.	VLADA	Mitochondrial fission process protein 1
649.	LADAL	Putative PIP5K1A and PSMD4-like protein
650.	LADAL	Mesogenin-1
651.	LADAL	Serpin E3 precursor
652.	LADAL	Tudor domain-containing protein 15
653.	LADAL	Mediator of RNA polymerase II transcription subunit 24
654.	LADAL	Transforming acidic coiled-coil-containing protein 2
655.	LADAL	Prelamin-A/C precursor
656.	LADAL	Uridine 5′-monophosphate synthase
657.	LADAL	Microtubule-associated protein 4
658.	LADAL	Cytosol aminopeptidase
659.	LADAL	Ubiquitin carboxyl-terminal hydrolase 6
660.	LADAL	Mu-type opioid receptor

(*Continued*)

 © 2023. The Author(s).

Supplementary Table S1 (*Continued*)

661.	LADAL	Delta-type opioid receptor
662.	LADAL	Kappa-type opioid receptor
663.	LADAL	UDP-glucuronosyltransferase 2B15 precursor
664.	LADAL	26S proteasome non-ATPase regulatory subunit 4
665.	LADAL	Glycoprotein Xg precursor
666.	LADAL	NACHT, LRR, and PYD domains-containing protein 11
667.	LADAL	40S ribosomal protein S15a
668.	LADAL	Interferon-related developmental regulator 2
669.	LADAL	DnaJ homolog subfamily C member 3 precursor
670.	LADAL	Plastin-1
671.	LADAL	CST complex subunit CTC1
672.	LADAL	Autophagy-related protein 2 homolog A
673.	LADAL	Speriolin
674.	LADAL	NLR family CARD domain-containing protein 3
675.	LADAL	Protein SCAI
676.	LADAL	E3 ubiquitin-protein ligase ZNRF1
677.	LADAL	MAPK-interacting and spindle-stabilizing protein-like
678.	LADAL	Ubiquitin carboxyl-terminal hydrolase 32 precursor
679.	LADAL	CD99 antigen-like protein 2 precursor
680.	LADAL	Gamma-secretase subunit APH-1A
681.	LADAL	Aurora kinase B
682.	LADAL	Methionine–tRNA ligase, mitochondrial precursor
683.	LADAL	Galactose-3-O-sulfotransferase 4
684.	LADAL	Phosphatidylinositol N-acetylglucosaminyl transferase subunit Q
685.	LADAL	Membrane-associated phosphatidylinositol transfer protein 3
686.	LADAL	Ras-related protein Rab-34
687.	LADAL	Cilia- and flagella-associated protein 74
688.	LADAL	Tetratricopeptide repeat protein 12
689.	LADAL	Aurora kinase C
690.	LADAL	Nucleotide-binding oligomerization domain-containing protein 1
691.	ADALT	Transmembrane protein 8B
692.	ADALT	Microtubule-associated protein 4
693.	ADALT	NACHT, LRR, and PYD domains-containing protein 11
694.	ADALT	Hemoglobin subunit alpha
695.	ADALT	Probable methyltransferase TARBP1
696.	ADALT	Ral GTPase-activating protein subunit beta
697.	ADALT	C-type lectin domain family 4 member F
698.	ADALT	Aurora kinase C
699.	ADALT	Plakophilin-3
700.	DALTA	Alpha-fetoprotein precursor
701.	DALTA	Nuclear pore complex protein Nup107
702.	DALTA	Di-N-acetyl chitobiose precursor
703.	DALTA	Ral guanine nucleotide dissociation stimulator
704.	DALTA	E3 ubiquitin-protein ligase HUWE1

 © 2023. The Author(s).

Supplementary Table S1 (*Continued*)

705.	DALTA	Spindle and centriole-associated protein 1
706.	DALTA	Zinc finger protein 653
707.	DALTA	Membrane-associated phosphatidylinositol transfer protein 2
708.	DALTA	Phosphatidylinositol glycan anchor biosynthesis class U protein
709.	DALTA	Calcyclin-binding protein
710.	DALTA	Rho GTPase-activating protein 35
711.	DALTA	Chromodomain Y-like protein
712.	ALTAP	Maestro heat-like repeat-containing protein family member 6
713.	ALTAP	Leucine-rich repeat-containing protein 37A2 precursor
714.	ALTAP	Leucine-rich repeat-containing protein 37A precursor
715.	ALTAP	Proline-rich protein 33
716.	ALTAP	Leucine-rich repeat-containing protein 37A3 precursor
717.	ALTAP	GDH/6PGL endoplasmic bifunctional protein precursor
718.	ALTAP	Gastrin/cholecystokinin type B receptor
719.	ALTAP	Amiloride-sensitive sodium channel subunit alpha
720.	ALTAP	Isocitrate dehydrogenase [NADP], mitochondrial precursor
721.	ALTAP	Sonic hedgehog protein precursor
722.	ALTAP	Glutamate-rich protein 3
723.	ALTAP	Coiled-coil domain-containing protein 149
724.	ALTAP	Progestin and adipoQ receptor family member 4
725.	ALTAP	AT-rich interactive domain-containing protein 1B
726.	ALTAP	ATP-binding cassette subfamily A member 10
727.	ALTAP	Nucleolar complex protein 4 homolog
728.	ALTAP	RING finger protein 208
729.	ALTAP	RING finger protein 39
730.	ALTAP	Akirin-1
731.	LTAPL	Olfactory receptor 4N4C
732.	LTAPL	Solute carrier family 22 member 20
733.	LTAPL	Circadian locomotor output cycles protein kaput
734.	LTAPL	Promotilin precursor
735.	LTAPL	Iroquois-class homeodomain protein IRX-4
736.	LTAPL	Beclin-1
737.	LTAPL	Serine/threonine-protein phosphatase 4 regulatory subunit 3B
738.	LTAPL	Serine/threonine-protein phosphatase 4 regulatory subunit 3A
739.	LTAPL	Extracellular matrix protein FRAS1 precursor
740.	LTAPL	Major facilitator superfamily domain-containing protein 6-like
741.	LTAPL	Olfactory receptor 4N5
742.	LTAPL	Doublesex- and mab-3-related transcription factor C2
743.	LTAPL	Olfactory receptor 4N4
744.	LTAPL	Olfactory receptor 4N2
745.	LTAPL	Solute carrier family 25 member 43
746.	LTAPL	CKLF-like MARVEL transmembrane domain-containing protein 3
747.	LTAPL	Epithelial splicing regulatory protein 2
748.	LTAPL	5′ exonuclease Apollo

(*Continued*)

 © 2023. The Author(s).

Supplementary Table S1 (*Continued*)

749.	LTAPL	Midasin
750.	LTAPL	Protocadherin beta-12 precursor
751.	TAPLD	Histone-lysine N-methyltransferase 2D
752.	TAPLD	Nebulin
753.	TAPLD	Elongation factor Tu, mitochondrial precursor
754.	TAPLD	Calcium-binding mitochondrial carrier protein SCaMC-2
755.	TAPLD	Calcium-binding mitochondrial carrier protein SCaMC-1
756.	TAPLD	Interleukin-27 receptor subunit alpha precursor
757.	TAPLD	Transmembrane protein 150A
758.	TAPLD	Protein FAM71E2
759.	TAPLD	Solute carrier family 25 member 41
760.	TAPLD	Calcium-binding mitochondrial carrier protein SCaMC-3
761.	TAPLD	Protocadherin beta-12 precursor
762.	APLDH	Maltase-glucoamylase, intestinal
763.	APLDH	Probable maltase-glucoamylase 2
764.	APLDH	Protein phosphatase 1 regulatory subunit 16A precursor
765.	APLDH	Ubiquitin carboxyl-terminal hydrolase 47
766.	APLDH	Aryl hydrocarbon receptor nuclear translocator 2
767.	PLDHK	6-Pyruvoyl tetrahydrobiopterin synthase
768.	LDHKD	Leucine-rich repeat-containing protein 52 precursor
769.	LDHKD	Histone deacetylase complex subunit SAP130
770.	LDHKD	Protein FAM234A
771.	HKDKS	Protein O-mannosyl-transferase TMTC4
772.	HKDKS	Nucleoporin NUP35
773.	HKDKS	Histone deacetylase 7
774.	KDKSL	Transcription factor E2F8
775.	KDKSL	Rho-related GTP-binding protein RhoD precursor
776.	KDKSL	Probable ubiquitin carboxyl-terminal hydrolase FAF-Y
777.	KDKSL	Bestrophin-1
778.	KDKSL	Nodal modulator 3 precursor
779.	KDKSL	Glutamate receptor ionotropic, NMDA 2B precursor
780.	KDKSL	Protein Shroom2
781.	KDKSL	Nodal modulator 1 precursor
782.	KDKSL	Nodal modulator 2 precursor
783.	KDKSL	Protein O-mannosyl-transferase TMTC4
784.	KDKSL	Probable ubiquitin carboxyl-terminal hydrolase FAF-X
785.	KDKSL	Transcription factor E2F7
786.	KDKSL	Neuromedin-U receptor 2
787.	KDKSL	A disintegrin and metalloproteinase with thrombospondin motifs 5 precursor
788.	DKSLQ	Choline O-acetyltransferase
789.	DKSLQ	StAR-related lipid transfer protein 6
790.	DKSLQ	Glutamate receptor ionotropic, NMDA 2B precursor
791.	DKSLQ	Rho-associated protein kinase 1
792.	DKSLQ	Taperin

Supplementary Table S1 (*Continued*)

793.	DKSLQ	Coiled-coil domain-containing protein 181
794.	DKSLQ	Pikachurin precursor
795.	DKSLQ	Serpin A11 precursor
796.	DKSLQ	GATOR complex protein NPRL2
797.	DKSLQ	Protocadherin Fat 2 precursor
798.	DKSLQ	Unconventional myosin-Vb
799.	DKSLQ	Brefeldin A-inhibited guanine nucleotide-exchange protein 1
800.	KSLQS	Potassium voltage-gated channel subfamily KQT member 2
801.	KSLQS	Myosin-7
802.	KSLQS	Myosin-6
803.	KSLQS	Neurotensin receptor type 1
804.	KSLQS	Utrophin
805.	KSLQS	Transcription factor RFX3
806.	KSLQS	Microtubule-associated protein 1A
807.	KSLQS	Zinc finger protein Rlf
808.	KSLQS	Inactive tyrosine-protein kinase 7 precursor
809.	KSLQS	Pro-interleukin-16
810.	KSLQS	Signal transducer and activator of transcription 4
811.	KSLQS	Peroxisomal acyl-coenzyme A oxidase 1
812.	KSLQS	Occludin
813.	KSLQS	Probable tubulin polyglutamylase TTLL9
814.	KSLQS	Leucine-rich repeat-containing protein 66
815.	KSLQS	Zinc finger protein 773
816.	KSLQS	Synaptonemal complex protein 3
817.	KSLQS	Enoyl-CoA hydratase domain-containing protein 3, mitochondrial precursor
818.	KSLQS	Zinc finger protein 419
819.	KSLQS	Centrosomal protein of 72 kDa
820.	KSLQS	Sodium channel protein type 10 subunit alpha
821.	SLQSL	TLD domain-containing protein 2
822.	SLQSL	Inhibitor of nuclear factor kappa-B kinase subunit beta
823.	SLQSL	Glutamate receptor ionotropic, delta-2 precursor
824.	SLQSL	Leucine-rich repeat-containing G-protein coupled receptor 5 precursor
825.	SLQSL	Ubiquitin carboxyl-terminal hydrolase 1
826.	SLQSL	Ataxin-1-like
827.	SLQSL	Myosin-7
828.	SLQSL	Myosin-6
829.	SLQSL	Homeobox protein Hox-B3
830.	SLQSL	Plasma membrane calcium-transporting ATPase 4
831.	SLQSL	Neurotensin receptor type 1
832.	SLQSL	Transcription factor HIVEP2
833.	SLQSL	Homeobox protein cut-like 1
834.	SLQSL	Transcription factor RFX3
835.	SLQSL	Protein AF-10
836.	SLQSL	Homeobox protein PKNOX1

(*Continued*)

Supplementary Table S1 (*Continued*)

837.	SLQSL	Coronin-7
838.	SLQSL	DENN domain-containing protein 2B
839.	SLQSL	Tastin
840.	SLQSL	Metal regulatory transcription factor 1
841.	SLQSL	Coiled-coil domain-containing protein 14
842.	SLQSL	Rho GTPase-activating protein 29
843.	SLQSL	Coiled-coil domain-containing protein 18
844.	SLQSL	Protein FAM222A
845.	SLQSL	Uncharacterized protein KIAA1614
846.	SLQSL	Leucine-rich repeat-containing protein 66
847.	SLQSL	WD repeat- and FYVE domain-containing protein 4
848.	SLQSL	Peroxiredoxin-like 2C
849.	SLQSL	Retinoic acid-induced protein 1
850.	SLQSL	Alpha-protein kinase 2
851.	SLQSL	DNA damage-induced apoptosis suppressor protein
852.	SLQSL	RNA exonuclease 1 homolog
853.	SLQSL	Capping protein, Arp2/3 and myosin-I linker protein 3
854.	SLQSL	Endonuclease 8-like 2
855.	SLQSL	Exocyst complex component 2
856.	SLQSL	t-SNARE domain-containing protein 1
857.	SLQSL	Transient receptor potential cation channel subfamily M member 7
858.	SLQSL	Mitogen-activated protein kinase kinase kinase 5
859.	SLQSL	Telomerase protein component 1
860.	SLQSL	Leucine-rich repeat-containing protein 1
861.	SLQSL	Hyccin
862.	SLQSL	Histone-lysine N-methyltransferase SETD2
863.	SLQSL	Leucine-rich repeat-containing G-protein coupled receptor 6 precursor
864.	SLQSL	Alpha-N-acetylgalactosaminidase alpha-2,6-sialyltransferase 1
865.	SLQSL	Tropomodulin-4
866.	SLQSL	Tuftelin-interacting protein 11
867.	SLQSL	DNA mismatch repair protein Mlh3
868.	SLQSL	ALK tyrosine kinase receptor precursor
869.	SLQSL	Structural maintenance of chromosomes protein 3
870.	SLQSL	Disks large-associated protein 4
871.	LQSLT	Putative serine protease 46
872.	LQSLT	C-X-C chemokine receptor type 6
873.	LQSLT	Zinc finger E-box-binding homeobox 2
874.	LQSLT	Tudor domain-containing protein 6
875.	LQSLT	Lipoprotein lipase precursor
876.	LQSLT	Glial fibrillary acidic protein
877.	LQSLT	Desmoplakin
878.	LQSLT	Condensin-2 complex subunit D3
879.	LQSLT	Protein FAM160A1
880.	LQSLT	Activated CDC42 kinase 1

 © 2023. The Author(s).

Supplementary Table S1 (*Continued*)

881.	LQSLT	Dynein heavy chain 14, axonemal
882.	LQSLT	Proteasome activator complex subunit 4
883.	LQSLT	T-cell immunoreceptor with Ig and ITIM domains precursor
884.	LQSLT	Uncharacterized protein KIAA1755
885.	LQSLT	Thyroid adenoma-associated protein
886.	LQSLT	Retinoic acid-induced protein 1
887.	LQSLT	Neuron navigator 3
888.	LQSLT	Neuron navigator 2
889.	LQSLT	SLIT and NTRK-like protein 4 precursor
890.	LQSLT	Dedicator of cytokinesis protein 3
891.	LQSLT	Bestrophin-3
892.	LQSLT	Histone-lysine N-methyltransferase 2C
893.	LQSLT	Connector enhancer of kinase suppressor of ras 1
894.	LQSLT	Mitochondrial import receptor subunit TOM40B
895.	LQSLT	Sperm-associated antigen 5
896.	LQSLT	Mediator of RNA polymerase II transcription subunit 15
897.	LQSLT	Solute carrier family 12 member 5
898.	LQSLT	Leucine-rich repeat-containing protein 40
899.	LQSLT	Solute carrier family 12 member 6
900.	LQSLT	Neurexin-1 precursor
901.	LQSLT	Glutamate receptor ionotropic, delta-1 precursor
902.	LQSLT	Solute carrier family 12 member 4
903.	LQSLT	Inactive phospholipase C-like protein 2
904.	LQSLT	Solute carrier family 12 member 7
905.	QSLTL	T cell receptor beta variable 29-1 precursor
906.	QSLTL	Lysine-specific demethylase 4E
907.	QSLTL	Arf-GAP with SH3 domain, ANK repeat, and PH domain-containing protein 2
908.	QSLTL	Leukocyte immunoglobulin-like receptor subfamily B member 3 precursor
909.	QSLTL	Tripartite motif-containing protein 3
910.	QSLTL	T-cell surface glycoprotein CD4 precursor
911.	QSLTL	Centromere-associated protein E precursor
912.	QSLTL	Zinc finger protein 91
913.	QSLTL	Zinc finger protein 273
914.	QSLTL	Leukocyte immunoglobulin-like receptor subfamily A member 6 precursor
915.	QSLTL	Voltage-dependent calcium channel subunit alpha-2/delta-4 precursor
916.	QSLTL	Coiled-coil domain-containing protein 33
917.	QSLTL	Hemicentin-2 precursor
918.	QSLTL	Arf-GAP with SH3 domain, ANK repeat and PH domain-containing protein 3
919.	QSLTL	Leucine-rich repeat-containing protein 45
920.	QSLTL	Zinc finger protein 681
921.	QSLTL	Sperm-associated antigen 5
922.	QSLTL	Centrosomal protein of 89 kDa
923.	QSLTL	Zinc finger protein 644
924.	QSLTL	Sialate O-acetylesterase precursor

(*Continued*)

 © 2023. The Author(s).

Supplementary Table S1 (*Continued*)

925.	QSLTL	DNA damage-inducible transcript 4 protein
926.	QSLTL	Centlein
927.	QSLTL	Axin-2
928.	SLTLD	Arf-GAP with SH3 domain, ANK repeat, and PH domain-containing protein 2
929.	SLTLD	Cytochrome P450 11B1, mitochondrial precursor
930.	SLTLD	Cytochrome P450 11B2, mitochondrial precursor
931.	SLTLD	Vascular cell adhesion protein 1 precursor
932.	SLTLD	Tyrosine-protein phosphatase non-receptor type 5
933.	SLTLD	Coronin-7
934.	SLTLD	Solute carrier family 35 member B1
935.	SLTLD	BCL2/adenovirus E1B 19 kDa protein-interacting protein 2
936.	SLTLD	Next to BRCA1 gene 1 protein
937.	SLTLD	Arf-GAP with coiled-coil, ANK repeat, and PH domain-containing protein 1
938.	SLTLD	Arf-GAP with coiled-coil, ANK repeat, and PH domain-containing protein 2
939.	SLTLD	Receptor-type tyrosine-protein phosphatase R precursor
940.	SLTLD	Olfactory receptor 6N2
941.	SLTLD	Arf-GAP with SH3 domain, ANK repeat, and PH domain-containing protein 3
942.	SLTLD	Placenta-specific protein 4
943.	SLTLD	Arf-GAP with coiled-coil, ANK repeat, and PH domain-containing protein 3
944.	SLTLD	FH2 domain-containing protein 1
945.	SLTLD	Lysine-specific demethylase 5B
946.	SLTLD	Serine/arginine repetitive matrix protein 2
947.	LTLDQ	Uncharacterized protein C2orf78
948.	LTLDQ	Tryptophan–tRNA ligase, cytoplasmic
949.	LTLDQ	Forkhead box protein M1
950.	LTLDQ	tRNA methyltransferase 10 homolog C precursor
951.	LTLDQ	TRAF family member-associated NF-kappa-B activator
952.	LTLDQ	Interleukin-12 receptor subunit beta-2 precursor
953.	LTLDQ	Myocilin precursor
954.	LTLDQ	NmrA-like family domain-containing protein 1
955.	LTLDQ	Serine/arginine repetitive matrix protein 2
956.	TLDQS	Aminopeptidase N
957.	TLDQS	Interleukin-12 subunit beta precursor
958.	TLDQS	Serine/threonine-protein kinase mTOR
959.	TLDQS	Armadillo repeat-containing X-linked protein 4
960.	TLDQS	Ovostatin homolog 2 precursor
961.	TLDQS	Ovostatin homolog 1 precursor
962.	TLDQS	Adhesion G protein-coupled receptor G3 precursor
963.	TLDQS	Ferredoxin-fold anticodon-binding domain-containing protein 1
964.	TLDQS	PH and SEC7 domain-containing protein 3
965.	TLDQS	Serine/arginine repetitive matrix protein 2
966.	TLDQS	Calcineurin-binding protein cabin-1
967.	LDQSV	Transcription factor SOX-30
968.	LDQSV	Intestinal-type alkaline phosphatase precursor

 © 2023. The Author(s).

Supplementary Table S1 (*Continued*)

969.	LDQSV	Transcription factor AP-2-epsilon
970.	LDQSV	Transcription factor AP-2-beta
971.	LDQSV	Myelin regulatory factor-like protein
972.	LDQSV	Killer cell immunoglobulin-like receptor 2DL4 precursor
973.	LDQSV	Lysine-specific demethylase 5D
974.	LDQSV	Oxysterol-binding protein-related protein 5
975.	LDQSV	DNA repair protein REV1
976.	LDQSV	2'-5′-Oligoadenylate synthase 3
977.	DQSVR	Transducin beta-like protein 3
978.	DQSVR	Striated muscle preferentially expressed protein kinase
979.	QSVRK	RNA/RNP complex-1-interacting phosphatase
980.	QSVRK	Mucin-19 precursor
981.	QSVRK	Protein ELYS
982.	QSVRK	TBC1 domain family member 14
983.	SVRKN	Hepatocyte nuclear factor 4-alpha
984.	SVRKN	Nuclear receptor subfamily 2 group C member 2
985.	SVRKN	DNA-binding protein inhibitor ID-2
986.	SVRKN	Serine/threonine-protein phosphatase 4 regulatory subunit 4
987.	SVRKN	Cytokine-dependent hematopoietic cell linker
988.	SVRKN	Symplekin
989.	SVRKN	Cilia- and flagella-associated protein 300
990.	SVRKN	E3 ubiquitin-protein ligase TRIM31
991.	VRKNE	Leucine-rich repeat serine/threonine-protein kinase 1
992.	VRKNE	Protein disulfide isomerase CRELD2 precursor
993.	VRKNE	Receptor-type tyrosine-protein phosphatase H precursor
994.	RKNEK	1-phosphatidylinositol 4,5-bisphosphate phosphodiesterase eta-2
995.	RKNEK	ATP-dependent Clp protease ATP-binding subunit clpX-like, mitochondrial precursor
996.	RKNEK	DNA replication licensing factor MCM3
997.	RKNEK	Peptidyl-prolyl cis-trans isomerase G
998.	RKNEK	BRISC complex subunit Abraxas 2
999.	RKNEK	Uncharacterized protein C10orf120
1000.	RKNEK	Terminal uridylyltransferase 7
1001.	RKNEK	Coiled-coil domain-containing protein 186
1002.	RKNEK	GAS2-like protein 1
1003.	RKNEK	Caspase recruitment domain-containing protein 6
1004.	RKNEK	Midasin
1005.	RKNEK	Hepatoma-derived growth factor-related protein 3
1006.	KNEKL	Gamma-secretase-activating protein
1007.	KNEKL	PH and SEC7 domain-containing protein 1
1008.	KNEKL	U5 small nuclear ribonucleoprotein 200 kDa helicase
1009.	KNEKL	Lathosterol oxidase
1010.	KNEKL	Centromere-associated protein E precursor
1011.	KNEKL	Coiled-coil domain-containing protein 40
1012.	KNEKL	Transcription initiation factor TFIID subunit 7-like

(*Continued*)

Supplementary Table S1 (*Continued*)

1013.	KNEKL	Centrosomal protein of 135 kDa
1014.	KNEKL	Cytospin-A
1015.	KNEKL	Ral GTPase-activating protein subunit alpha-1
1016.	KNEKL	E3 ubiquitin-protein ligase RNF180
1017.	KNEKL	Histone-lysine N-methyltransferase KMT5C
1018.	KNEKL	Chondroitin sulfate N-acetylgalactosaminyltransferase 1
1019.	KNEKL	Phosphatase and actin regulator 3
1020.	KNEKL	PH and SEC7 domain-containing protein 2
1021.	KNEKL	Centromere protein K
1022.	KNEKL	PH and SEC7 domain-containing protein 3
1023.	KNEKL	Testin
1024.	KNEKL	Microtubule-associated tumor suppressor 1
1025.	KNEKL	Calcium-regulated heat-stable protein 1
1026.	KNEKL	Protein SGT1 homolog
1027.	NEKLK	Gamma-secretase-activating protein
1028.	NEKLK	Protein phosphatase 1 regulatory subunit 12A
1029.	NEKLK	Centrosomal protein of 290 kDa
1030.	NEKLK	DNA-directed RNA polymerases I and III subunit RPAC1
1031.	NEKLK	DNA polymerase theta
1032.	NEKLK	Nucleoprotein TPR
1033.	NEKLK	Synaptonemal complex protein 1
1034.	NEKLK	Protein dopey-1
1035.	NEKLK	E3 ubiquitin-protein ligase BRE1A
1036.	NEKLK	ATPase family AAA domain-containing protein 2
1037.	NEKLK	Gamma-glutamyl hydrolase precursor
1038.	NEKLK	Glomulin
1039.	NEKLK	Optineurin
1040.	NEKLK	Phosphatase and actin regulator 3
1041.	NEKLK	Centromere protein K
1042.	NEKLK	Serine/threonine-protein kinase 31
1043.	NEKLK	Myosin light chain kinase 2, skeletal/cardiac muscle
1044.	NEKLK	Leucine zipper transcription factor-like protein 1
1045.	NEKLK	B-cell receptor-associated protein 29
1046.	NEKLK	Microtubule-associated tumor suppressor 1
1047.	NEKLK	Myosin-4
1048.	EKLKL	Diphosphoinositol polyphosphate phosphohydrolase NUDT4B
1049.	EKLKL	Testis-expressed protein 52
1050.	EKLKL	UPF0600 protein C5orf51
1051.	EKLKL	26S proteasome non-ATPase regulatory subunit 12
1052.	EKLKL	Pescadillo homolog
1053.	EKLKL	Adhesion G protein-coupled receptor B1 precursor
1054.	EKLKL	Histone-lysine N-methyltransferase 2D
1055.	EKLKL	Integrin beta-1-binding protein 1
1056.	EKLKL	Period circadian protein homolog 2

 © 2023. The Author(s).

Supplementary Table S1 (*Continued*)

1057.	EKLKL	Keratin, type II cytoskeletal 8
1058.	EKLKL	Glutathione S-transferase Mu 1
1059.	EKLKL	Glucose-6-phosphate 1-dehydrogenase
1060.	EKLKL	Glutathione S-transferase Mu 5
1061.	EKLKL	Centromere protein F precursor
1062.	EKLKL	ATP-sensitive inward rectifier potassium channel 10
1063.	EKLKL	TBC1 domain family member 8B
1064.	EKLKL	Putative WASP homolog-associated protein with actin, membranes and microtubules-like protein 1
1065.	EKLKL	Coiled-coil domain-containing protein 40
1066.	EKLKL	Centrosomal protein of 162 kDa
1067.	EKLKL	TBC1 domain family member 9
1068.	EKLKL	Ankyrin repeat domain-containing protein 23
1069.	EKLKL	Serologically defined colon cancer antigen 8
1070.	EKLKL	Mediator of RNA polymerase II transcription subunit 12-like protein
1071.	EKLKL	Dynein heavy chain 10, axonemal
1072.	EKLKL	Neuron navigator 2
1073.	EKLKL	CLK4-associating serine/arginine rich protein
1074.	EKLKL	Cyclic GMP-AMP synthase
1075.	EKLKL	Centrosomal protein of 112 kDa
1076.	EKLKL	Myb/SANT-like DNA-binding domain-containing protein 4
1077.	EKLKL	Dedicator of cytokinesis protein 8
1078.	EKLKL	Diphosphoinositol polyphosphate phosphohydrolase 3-alpha
1079.	EKLKL	Protein Jade-3
1080.	EKLKL	Dynein heavy chain 11, axonemal
1081.	EKLKL	Diphosphoinositol polyphosphate phosphohydrolase 3-beta
1082.	EKLKL	Protein transport protein Sec16B
1083.	EKLKL	Testicular spindle-associated protein SHCBP1L
1084.	EKLKL	Golgi resident protein GCP60
1085.	EKLKL	Echinoderm microtubule-associated protein-like 4
1086.	EKLKL	Anoctamin-2
1087.	EKLKL	Intraflagellar transport protein 57 homolog
1088.	EKLKL	Diphosphoinositol polyphosphate phosphohydrolase 2
1089.	EKLKL	Tryptophan–tRNA ligase, mitochondrial precursor
1090.	EKLKL	Axin-2
1091.	EKLKL	Glypican-6 precursor
1092.	KLKLA	Adhesion G protein-coupled receptor B1 precursor
1093.	KLKLA	Centromere protein F precursor
1094.	KLKLA	SAP domain-containing ribonucleoprotein
1095.	KLKLA	Unhealthy ribosome biogenesis protein 2 homolog
1096.	KLKLA	Mitochondrial 10-formyltetrahydrofolate dehydrogenase
1097.	KLKLA	DDB1- and CUL4-associated factor 12-like protein 2
1098.	KLKLA	Peroxisomal N(1)-acetyl-spermine/spermidine oxidase
1099.	KLKLA	Ubinuclein-2
1100.	KLKLA	Dynein heavy chain 10, axonemal

(*Continued*)

 © 2023. The Author(s).

Supplementary Table S1 (*Continued*)

1101.	KLKLA	SURP and G-patch domain-containing protein 2
1102.	KLKLA	ADP-ribosylation factor-like protein 14
1103.	KLKLA	Plakophilin-2
1104.	KLKLA	Extended synaptotagmin-1
1105.	LKLAA	Aquaporin-12B
1106.	LKLAA	Rab GTPase-activating protein 1-like, isoform 10
1107.	LKLAA	NADH dehydrogenase [ubiquinone] 1 alpha subcomplex subunit 10, mitochondrial
1108.	LKLAA	Probable global transcription activator SNF2L1
1109.	LKLAA	Oxygen-regulated protein 1
1110.	LKLAA	Twist-related protein 1
1111.	LKLAA	Transcription factor E2F4
1112.	LKLAA	Drebrin
1113.	LKLAA	Tyrosine-protein phosphatase non-receptor type 21
1114.	LKLAA	DNA annealing helicase and endonuclease ZRANB3
1115.	LKLAA	Coiled-coil domain-containing protein 121
1116.	LKLAA	Na(+)/H(+) exchange regulatory cofactor NHE-RF4
1117.	LKLAA	Myosin light chain kinase family member 4
1118.	LKLAA	Aquaporin-12A
1119.	LKLAA	Divergent protein kinase domain 2A precursor
1120.	LKLAA	Twist-related protein 2
1121.	LKLAA	Protein FAM163A
1122.	LKLAA	FYVE, RhoGEF and PH domain-containing protein 4
1123.	LKLAA	Myosin light chain kinase 2, skeletal/cardiac muscle
1124.	LKLAA	Enhancer of polycomb homolog 1
1125.	LKLAA	ATP-dependent DNA helicase PIF1
1126.	LKLAA	Voltage-dependent T-type calcium channel subunit alpha-1I
1127.	LKLAA	Neurochondrin
1128.	LKLAA	Serine/threonine-protein kinase tousled-like 1
1129.	LKLAA	Transient receptor potential cation channel subfamily V member 2
1130.	KLAAQ	Aquaporin-12B
1131.	KLAAQ	Villin-like protein
1132.	KLAAQ	Adenomatous polyposis coli protein 2
1133.	KLAAQ	DNA-directed RNA polymerase II subunit GRINL1A
1134.	KLAAQ	Glycine amidinotransferase, mitochondrial precursor
1135.	KLAAQ	Enteropeptidase precursor
1136.	KLAAQ	ATP-dependent RNA helicase A
1137.	KLAAQ	Unconventional myosin-VIIb
1138.	KLAAQ	Alpha-protein kinase 2
1139.	KLAAQ	Aquaporin-12A
1140.	KLAAQ	Dynein heavy chain 3, axonemal
1141.	KLAAQ	Flagellum-associated coiled-coil domain-containing protein 1
1142.	KLAAQ	Tubulin-specific chaperone D
1143.	KLAAQ	Putative GRINL1B complex locus protein 2
1144.	KLAAQ	Guanylate-binding protein 3

 © 2023. The Author(s).

Supplementary Table S1 (*Continued*)

1145.	KLAAQ	Dynein heavy chain 9, axonemal
1146.	KLAAQ	Interleukin-37 precursor
1147.	LAAQG	Aquaporin-12B
1148.	LAAQG	DNA-binding protein RFXANK
1149.	LAAQG	Zinc finger MYND domain-containing protein 10
1150.	LAAQG	Dysferlin
1151.	LAAQG	Protein transport protein Sec31A
1152.	LAAQG	Fructose-bisphosphate aldolase C
1153.	LAAQG	Uroporphyrinogen-III synthase
1154.	LAAQG	Thymidine phosphorylase precursor
1155.	LAAQG	Potassium-transporting ATPase alpha chain 1
1156.	LAAQG	Acetylcholinesterase precursor
1157.	LAAQG	Macrosialin precursor
1158.	LAAQG	Glycine amidinotransferase, mitochondrial precursor
1159.	LAAQG	Receptor-interacting serine/threonine-protein kinase 4
1160.	LAAQG	Ubiquitin-protein ligase E3A
1161.	LAAQG	Sodium channel protein type 5 subunit alpha
1162.	LAAQG	Protein YIF1B
1163.	LAAQG	Polypeptide N-acetylgalactosaminyltransferase 18
1164.	LAAQG	Protocadherin Fat 4 precursor
1165.	LAAQG	Probable E3 ubiquitin-protein ligase DTX2
1166.	LAAQG	Aquaporin-12A
1167.	LAAQG	Ankyrin repeat domain-containing protein 29
1168.	LAAQG	NACHT, LRR and PYD domains-containing protein 7
1169.	LAAQG	Prolyl hydroxylase EGLN2
1170.	LAAQG	Cytosolic carboxypeptidase 4
1171.	LAAQG	BTB/POZ domain-containing protein KCTD15
1172.	LAAQG	Rho GTPase-activating protein 39
1173.	LAAQG	Mitochondrial glutamate carrier 1
1174.	LAAQG	Ankyrin repeat family A protein 2
1175.	LAAQG	Protein transport protein Sec31B
1176.	LAAQG	NACHT, LRR, and PYD domain-containing protein 2
1177.	LAAQG	Serine/threonine-protein kinase TAO2
1178.	LAAQG	E3 ubiquitin-protein ligase DTX4
1179.	LAAQG	Doublesex- and mab-3-related transcription factor 2
1180.	AAQGA	Agrin precursor
1181.	AAQGA	Secretory carrier-associated membrane protein 2
1182.	AAQGA	Acetylcholinesterase precursor
1183.	AAQGA	Nuclear pore complex protein Nup214
1184.	AAQGA	Hyaluronidase-1 precursor
1185.	AAQGA	Forkhead box protein L1
1186.	AAQGA	Kinesin-like protein KIF7
1187.	AAQGA	Proline-rich transmembrane protein 3 precursor
1188.	AAQGA	Adipolin precursor

(*Continued*)

Supplementary Table S1 (*Continued*)

1189.	AAQGA	Obscurin
1190.	AAQGA	E3 ubiquitin-protein ligase MIB1
1191.	AAQGA	Protein FAM9A
1192.	AAQGA	Tetratricopeptide repeat protein 9B
1193.	AAQGA	Lethal(3)malignant brain tumor-like protein 4
1194.	AAQGA	Secretory carrier-associated membrane protein 5
1195.	AAQGA	Phosphoethanolamine/phosphocholine phosphatase
1196.	AAQGA	Nesprin-2
1197.	AAQGA	Prolyl hydroxylase EGLN2
1198.	AAQGA	Histone acetyltransferase KAT8
1199.	AAQGA	TBC1 domain family member 2B
1200.	AQGAE	Ceroid-lipofuscinosis neuronal protein 5
1201.	AQGAE	Trifunctional purine biosynthetic protein adenosine-3
1202.	AQGAE	Cyclic nucleotide-gated cation channel beta-1
1203.	AQGAE	Extracellular matrix protein FRAS1 precursor
1204.	AQGAE	E3 ubiquitin-protein ligase MIB1
1205.	AQGAE	Mitogen-activated protein kinase kinase kinase 5
1206.	AQGAE	Oxysterol-binding protein-related protein 1
1207.	AQGAE	Potassium voltage-gated channel subfamily E regulatory beta subunit 5
1208.	AQGAE	Plasmolipin
1209.	AQGAE	Protocadherin beta-3 precursor
1210.	AQGAE	Protocadherin beta-2 precursor
1211.	QGAEK	Ubiquinone biosynthesis protein COQ9, mitochondrial precursor
1212.	QGAEK	Complement component C6 precursor
1213.	QGAEK	Interferon-induced GTP-binding protein Mx2
1214.	QGAEK	Putative deoxyribonuclease TATDN3
1215.	QGAEK	Protein ZGRF1
1216.	QGAEK	E3 ubiquitin-protein ligase MIB1
1217.	QGAEK	A-kinase anchor protein 9
1218.	QGAEK	CCR4-NOT transcription complex subunit 10
1219.	QGAEK	Solute carrier family 12 member 6
1220.	QGAEK	FK506-binding protein-like
1221.	QGAEK	G patch domain-containing protein 8
1222.	QGAEK	Solute carrier family 12 member 4
1223.	GAEKT	Uncharacterized protein C20orf204 precursor
1224.	GAEKT	Decapping and exoribonuclease protein
1225.	GAEKT	WAP four-disulfide core domain protein 2 precursor
1226.	GAEKT	Kelch domain-containing protein 4
1227.	GAEKT	A-kinase anchor protein 9
1228.	GAEKT	Transcription factor COE4
1229.	GAEKT	Protocadherin Fat 2 precursor
1230.	GAEKT	G patch domain-containing protein 8
1231.	GAEKT	Acrosomal protein KIAA1210
1232.	AEKTY	Pericentrin

Supplementary Table S1 (*Continued*)

1233.	AEKTY	5′-AMP-activated protein kinase subunit gamma-1
1234.	AEKTY	G-protein coupled receptor-associated protein LMBRD2
1235.	AEKTY	Zinc finger protein 57
1236.	AEKTY	Zinc finger protein 555
1237.	AEKTY	Ryanodine receptor 2
1238.	AEKTY	5′-AMP-activated protein kinase subunit gamma-2
1239.	EKTYG	Centrosomal protein 43
1240.	EKTYG	Cell division cycle protein 16 homolog
1241.	EKTYG	Elongin-C
1242.	EKTYG	Zinc finger protein 605
1243.	EKTYG	2-Phosphoxylose phosphatase 1
1244.	EKTYG	Protocadherin gamma-A4 precursor
1245.	EKTYG	Protocadherin gamma-A11 precursor
1246.	KTYGN	Spermidine synthase
1247.	KTYGN	Oxygen-regulated protein 1
1248.	KTYGN	Protocadherin gamma-A4 precursor
1249.	KTYGN	Protocadherin gamma-A11 precursor
1250.	TYGNG	Matrilysin precursor
1251.	TYGNG	Poly(U)-specific endoribonuclease precursor
1252.	TYGNG	Protein OS-9 precursor
1253.	TYGNG	Kielin/chordin-like protein precursor
1254.	YGNGD	Matrilysin precursor
1255.	YGNGD	Teneurin-4
1256.	YGNGD	N-acetyllactosaminide beta-1,6-N-acetylglucosaminyl-transferase
1257.	YGNGD	Endoplasmic reticulum lectin 1 precursor
1258.	GNGDS	Adhesion G protein-coupled receptor B1 precursor
1259.	GNGDS	Zinc finger protein 350
1260.	GNGDS	Cohesin subunit SA-3
1261.	NGDSL	Neuroblastoma-amplified sequence
1262.	NGDSL	Granule associated Rac and RHOG effector protein 1
1263.	NGDSL	Interferon-induced GTP-binding protein Mx2
1264.	NGDSL	Cytochrome b-c1 complex subunit 2, mitochondrial precursor
1265.	NGDSL	Trafficking protein particle complex subunit 10
1266.	NGDSL	Bromodomain and WD repeat-containing protein 3
1267.	NGDSL	TBC1 domain family member 9
1268.	NGDSL	Ubiquitin carboxyl-terminal hydrolase 45
1269.	NGDSL	Zinc finger homeobox protein 4
1270.	NGDSL	TBC1 domain family member 19
1271.	NGDSL	Histone-lysine N-methyltransferase, H3 lysine-36 specific
1272.	NGDSL	Caspase recruitment domain-containing protein 11
1273.	NGDSL	Cysteine-rich motor neuron 1 protein precursor
1274.	NGDSL	Cohesin subunit SA-3
1275.	NGDSL	Influenza virus NS1A-binding protein
1276.	GDSLN	Deformed epidermal autoregulatory factor 1 homolog

(*Continued*)

 © 2023. The Author(s).

Supplementary Table S1 (*Continued*)

1277.	GDSLN	Kelch-like protein 18
1278.	GDSLN	Transcriptional activator Myb
1279.	GDSLN	Protein Shroom2
1280.	GDSLN	Zinc finger protein 267
1281.	GDSLN	Zinc finger protein 544
1282.	GDSLN	Autophagy-related protein 2 homolog B
1283.	GDSLN	Cysteine-rich protein 2-binding protein
1284.	GDSLN	Bromodomain adjacent to zinc finger domain protein 2B
1285.	DSLNT	Plexin-B2 precursor
1286.	DSLNT	Deformed epidermal autoregulatory factor 1 homolog
1287.	DSLNT	Transcriptional activator Myb
1288.	DSLNT	Pyruvate carboxylase, mitochondrial precursor
1289.	DSLNT	RNA-binding protein 48
1290.	DSLNT	Activating transcription factor 7-interacting protein 2
1291.	DSLNT	Testis, prostate and placenta-expressed protein precursor
1292.	DSLNT	Serine/threonine-protein kinase ULK2
1293.	DSLNT	Nesprin-2
1294.	DSLNT	DNA repair and recombination protein RAD54-like
1295.	DSLNT	Kelch-like protein 29
1296.	DSLNT	E3 ubiquitin-protein ligase MARCHF7
1297.	SLNTG	TRAF-type zinc finger domain-containing protein 1
1298.	SLNTG	Disintegrin and metalloproteinase domain-containing protein 10 precursor
1299.	SLNTG	Calmodulin-regulated spectrin-associated protein 2
1300.	SLNTG	Mucin-16
1301.	LNTGK	Protein shisa-9 precursor
1302.	LNTGK	DNA-directed RNA polymerase III subunit RPC1
1303.	LNTGK	Dysferlin
1304.	LNTGK	DNA polymerase alpha catalytic subunit
1305.	LNTGK	Collagen alpha-3 (VI) chain precursor
1306.	LNTGK	Ubiquitin carboxyl-terminal hydrolase 29
1307.	LNTGK	Myoferlin
1308.	NTGKL	DNA-directed RNA polymerase III subunit RPC1
1309.	NTGKL	Striatin
1310.	NTGKL	Insulin-like growth factor-binding protein 2 precursor
1311.	NTGKL	Zinc finger protein 425
1312.	NTGKL	Ras-like protein family member 11A
1313.	NTGKL	Cilia- and flagella-associated protein 47
1314.	NTGKL	Dynein heavy chain 8, axonemal
1315.	NTGKL	Histone-lysine N-methyltransferase PRDM9
1316.	NTGKL	RNA polymerase II subunit A C-terminal domain phosphatase
1317.	TGKLK	Mediator of RNA polymerase II transcription subunit 24
1318.	TGKLK	NAD(P)H dehydrogenase [quinone] 1
1319.	TGKLK	Rod cGMP-specific 3′,5′-cyclic phosphodiesterase subunit alpha precursor
1320.	TGKLK	Laminin subunit alpha-2 precursor

Supplementary Table S1 (*Continued*)

1321.	TGKLK	RNA-binding protein FUS
1322.	TGKLK	Rod cGMP-specific 3′,5′-cyclic phosphodiesterase subunit beta precursor
1323.	TGKLK	Probable 28S rRNA (cytosine(4447)-C(5))-methyltransferase
1324.	TGKLK	Cytosolic purine 5′-nucleotidase
1325.	TGKLK	Cytoskeleton-associated protein 5
1326.	TGKLK	Armadillo repeat-containing protein 4
1327.	TGKLK	Disabled homolog 2-interacting protein
1328.	TGKLK	Inositol hexakisphosphate and diphosphoinositol-pentakisphosphate kinase 1
1329.	TGKLK	Extracellular sulfatase Sulf-2 precursor
1330.	TGKLK	Serine/threonine-protein kinase DCLK2
1331.	GKLKN	Nuclear valosin-containing protein-like
1332.	GKLKN	Protein transport protein Sec24D
1333.	GKLKN	Death-associated protein kinase 1
1334.	GKLKN	Protein transport protein Sec24C
1335.	GKLKN	Ubiquitin carboxyl-terminal hydrolase 46
1336.	GKLKN	E3 ubiquitin-protein ligase XIAP
1337.	GKLKN	Helicase-like transcription factor
1338.	GKLKN	Ryanodine receptor 3
1339.	GKLKN	Myomegalin
1340.	GKLKN	Cytoplasmic dynein 2 heavy chain 1
1341.	GKLKN	Zinc phosphodiesterase ELAC protein 1
1342.	KLKND	Zinc finger protein 75D
1343.	KLKND	DnaJ homolog subfamily C member 3 precursor
1344.	KLKND	Coiled-coil domain-containing protein 178
1345.	KLKND	Zinc finger protein 318
1346.	KLKND	Immunoglobulin superfamily member 22
1347.	KLKND	Kinetochore scaffold 1
1348.	KLKND	Zinc finger protein 75A
1349.	KLKND	Mediator of RNA polymerase II transcription subunit 15
1350.	KLKND	Remodeling and spacing factor 1
1351.	KLKND	Caspase recruitment domain-containing protein 11
1352.	KLKND	Zinc finger protein 608
1353.	KLKND	Unconventional myosin-Vb
1354.	KLKND	Beta/gamma crystallin domain-containing protein 1
1355.	LKNDK	Sarcoplasmic/endoplasmic reticulum calcium ATPase 1
1356.	LKNDK	Cyclin-dependent kinase 13
1357.	LKNDK	Coiled-coil domain-containing protein 178
1358.	LKNDK	Cilia- and flagella-associated protein 47
1359.	LKNDK	Kinetochore scaffold 1
1360.	LKNDK	Equatorin precursor
1361.	LKNDK	Transcription factor 20
1362.	NDKVS	Mucin-2 precursor
1363.	NDKVS	Piezo-type mechanosensitive ion channel component 2
1364.	DKVSR	Microtubule-associated protein 1B

(*Continued*)

Supplementary Table S1 (*Continued*)

1365.	DKVSR	Probable E3 ubiquitin-protein ligase HECTD2
1366.	DKVSR	Polyribonucleotide nucleotidyltransferase 1, mitochondrial precursor
1367.	DKVSR	Dynein heavy chain 9, axonemal
1368.	KVSRF	E3 ubiquitin-protein ligase RNF103
1369.	KVSRF	Solute carrier family 46 member 3 precursor
1370.	KVSRF	Signal peptide, CUB and EGF-like domain-containing protein 1 precursor
1371.	KVSRF	Proline-rich protein 14
1372.	KVSRF	Uncharacterized protein C19orf44
1373.	KVSRF	Signal peptide, CUB and EGF-like domain-containing protein 2 precursor
1374.	VSRFD	Neurofibromin
1375.	VSRFD	Neurabin-2
1376.	SRFDF	Exportin-5
1377.	SRFDF	Unconventional myosin-X
1378.	RFDFI	Unconventional myosin-X
1379.	DFIRQ	Rho GTPase-activating protein 24
1380.	FIRQI	Dyslexia-associated protein KIAA0319-like protein
1381.	FIRQI	Eukaryotic translation initiation factor 3 subunit L
1382.	IRQIE	Spectrin beta chain, erythrocytic
1383.	IRQIE	Spectrin beta chain, non-erythrocytic 1
1384.	IRQIE	LisH domain-containing protein ARMC9
1385.	IRQIE	THO complex subunit 1
1386.	IRQIE	Leucine-rich repeat-containing protein 4 precursor
1387.	RQIEV	Protein ITPRID2
1388.	RQIEV	TBC domain-containing protein kinase-like protein
1389.	RQIEV	Leucine-rich repeat-containing protein 4 precursor
1390.	QIEVD	TBC domain-containing protein kinase-like protein
1391.	IEVDG	Rho-related GTP-binding protein RhoC precursor
1392.	IEVDG	Fibrocystin precursor
1393.	IEVDG	Netrin receptor DCC precursor
1394.	IEVDG	Ras-related protein Rab-15
1395.	IEVDG	Transforming protein RhoA precursor
1396.	IEVDG	Rho-related GTP-binding protein RhoB precursor
1397.	IEVDG	Sterol 26-hydroxylase, mitochondrial precursor
1398.	IEVDG	Piwi-like protein 3
1399.	IEVDG	Extracellular sulfatase Sulf-2 precursor
1400.	IEVDG	Cell migration-inducing and hyaluronan-binding protein precursor
1401.	IEVDG	Ribulose-phosphate 3-epimerase
1402.	IEVDG	NADPH oxidase 1
1403.	EVDGQ	Protein BNIP5
1404.	EVDGQ	RNA cytosine C(5)-methyltransferase NSUN2
1405.	EVDGQ	Interleukin enhancer-binding factor 3
1406.	EVDGQ	WD repeat-containing protein 44
1407.	EVDGQ	CapZ-interacting protein
1408.	EVDGQ	Disks large homolog 3

Supplementary Table S1 (*Continued*)

1409.	EVDGQ	Spermatid perinuclear RNA-binding protein
1410.	EVDGQ	Syntenin-2
1411.	EVDGQ	Probable RNA-binding protein 19
1412.	VDGQL	Putative serine protease 47 precursor
1413.	VDGQL	Protein BNIP5
1414.	VDGQL	Ataxin-1-like
1415.	VDGQL	Contactin-associated protein 1 precursor
1416.	VDGQL	Laminin subunit alpha-3 precursor
1417.	VDGQL	Secreted frizzled-related protein 5 precursor
1418.	VDGQL	E3 ubiquitin-protein ligase RNF146
1419.	VDGQL	Teneurin-3
1420.	VDGQL	Protein sel-1 homolog 1 precursor
1421.	VDGQL	Serine/threonine-protein phosphatase 6 regulatory subunit 1
1422.	DGQLI	U4/U6 small nuclear ribonucleoprotein Prp4
1423.	DGQLI	Presenilin-1
1424.	DGQLI	Cleavage stimulation factor subunit 1
1425.	DGQLI	Extracellular matrix protein FRAS1 precursor
1426.	DGQLI	Hemicentin-1 precursor
1427.	DGQLI	E3 ubiquitin-protein ligase RNF216
1428.	GQLIT	Peptidyl-prolyl cis-trans isomerase FKBP9 precursor
1429.	GQLIT	Nuclear transcription factor Y subunit alpha
1430.	GQLIT	Teneurin-2
1431.	QLITL	FRAS1-related extracellular matrix protein 3 precursor
1432.	QLITL	Centromere-associated protein E precursor
1433.	QLITL	GTPase-activating protein and VPS9 domain-containing protein 1
1434.	QLITL	Neuropeptide S receptor
1435.	QLITL	Phosphoinositide 3-kinase adapter protein 1
1436.	QLITL	Dynein regulatory complex protein 11
1437.	QLITL	Protein unc-13 homolog C
1438.	QLITL	Talin-2
1439.	LITLE	Bifunctional 3′-phosphoadenosine 5′-phosphosulfate synthase 1
1440.	LITLE	Protein disulfide-isomerase precursor
1441.	LITLE	Interferon-induced GTP-binding protein Mx1
1442.	LITLE	Cadherin-13 precursor
1443.	LITLE	AP-1 complex subunit sigma-2
1444.	LITLE	AP-1 complex subunit sigma-1A
1445.	LITLE	Methylmalonate-semialdehyde dehydrogenase [acylating], mitochondrial precursor
1446.	LITLE	Otoancorin precursor
1447.	LITLE	Xylosyltransferase 1
1448.	LITLE	Transmembrane protein 229B
1449.	LITLE	VPS10 domain-containing receptor SorCS1 precursor
1450.	LITLE	Vacuolar-sorting protein SNF8
1451.	LITLE	Cyclin-L2
1452.	LITLE	S-methylmethionine–homocysteine S-methyltransferase BHMT2

(*Continued*)

 © 2023. The Author(s).

Supplementary Table S1 (*Continued*)

1453.	LITLE	Protein unc-79 homolog
1454.	ITLES	Pre-mRNA-processing factor 40 homolog A
1455.	ITLES	NADH-cytochrome b5 reductase 3
1456.	ITLES	Calcium-activated potassium channel subunit alpha-1
1457.	ITLES	Protein FAM214A
1458.	ITLES	Pre-mRNA-processing factor 40 homolog B
1459.	ITLES	Tubulin–tyrosine ligase
1460.	ITLES	Zinc finger CCCH domain-containing protein 15
1461.	ITLES	Protein ELYS
1462.	ITLES	Tetratricopeptide repeat protein 17
1463.	TLESG	Ubiquitin carboxyl-terminal hydrolase 27
1464.	TLESG	Sialic acid-binding Ig-like lectin 6 precursor
1465.	TLESG	Tubby protein homolog
1466.	TLESG	DNA repair protein XRCC4
1467.	TLESG	Ubiquitin carboxyl-terminal hydrolase 51
1468.	TLESG	Nesprin-2
1469.	TLESG	Cationic amino acid transporter 3
1470.	TLESG	Titin
1471.	TLESG	F-box/WD repeat-containing protein 7
1472.	TLESG	Sialic acid-binding Ig-like lectin 12 precursor
1473.	TLESG	Sialic acid-binding Ig-like lectin 8 precursor
1474.	TLESG	Ubiquitin carboxyl-terminal hydrolase 22
1475.	TLESG	Sialic acid-binding Ig-like lectin 7 precursor
1476.	TLESG	Sialic acid-binding Ig-like lectin 9 precursor
1477.	LESGE	Putative ATP-dependent RNA helicase DDX11-like protein 8
1478.	LESGE	Zinc finger and BTB domain-containing protein 11
1479.	LESGE	Acrosomal protein SP-10 precursor
1480.	LESGE	5-hydroxytryptamine receptor 1F
1481.	LESGE	Protein EFR3 homolog A
1482.	LESGE	Rho GTPase-activating protein 19
1483.	LESGE	Sushi, von Willebrand factor type A, EGF and pentraxin domain-containing protein 1 precursor
1484.	LESGE	DDB1- and CUL4-associated factor 15
1485.	LESGE	Cyclic AMP-responsive element-binding protein 3-like protein 2
1486.	LESGE	AT-rich interactive domain-containing protein 3B
1487.	LESGE	E3 ubiquitin-protein ligase UBR1
1488.	LESGE	Putative ATP-dependent RNA helicase DDX12
1489.	LESGE	ATP-dependent DNA helicase DDX11
1490.	LESGE	PiggyBac transposable element-derived protein 1
1491.	LESGE	N-acetylneuraminate 9-O-acetyltransferase
1492.	LESGE	Tether containing UBX domain for GLUT4
1493.	LESGE	S-methylmethionine–homocysteine S-methyltransferase BHMT2
1494.	LESGE	Paladin
1495.	LESGE	RING finger protein 112
1496.	ESGEF	Rho GTPase-activating protein 19

 © 2023. The Author(s).

Supplementary Table S1 (*Continued*)

1497.	ESGEF	Telomerase protein component 1
1498.	ESGEF	Myosin-2
1499.	ESGEF	Exonuclease 1
1500.	ESGEF	Myosin-15
1501.	ESGEF	Disheveled-associated activator of morphogenesis 1
1502.	ESGEF	Myosin-4
1503.	SGEFQ	Ribonucleoside-diphosphate reductase large subunit
1504.	SGEFQ	G2/M phase-specific E3 ubiquitin-protein ligase
1505.	SGEFQ	Probable helicase senataxin
1506.	SGEFQ	Pecanex-like protein 1
1507.	GEFQV	Pantetheinase precursor
1508.	GEFQV	Paladin
1509.	EFQVY	Tudor domain-containing protein 15
1510.	FQVYK	Cytochrome c1, heme protein, mitochondrial precursor
1511.	FQVYK	Protein transport protein Sec16B
1512.	QVYKQ	NUAK family SNF1-like kinase 1
1513.	QVYKQ	Cytochrome c1, heme protein, mitochondrial precursor
1514.	QVYKQ	TBCC domain-containing protein 1
1515.	VYKQS	Ornithine carbamoyltransferase, mitochondrial precursor
1516.	VYKQS	Breast cancer type 1 susceptibility protein
1517.	VYKQS	Nck-associated protein 1
1518.	KQSHS	Putative aldo-keto reductase family 1 member C8
1519.	KQSHS	Protocadherin-15 precursor
1520.	KQSHS	Histone-lysine N-methyltransferase SETD2
1521.	QSHSA	DNA-directed RNA polymerase II subunit RPB9
1522.	QSHSA	Zinc finger homeobox protein 3
1523.	QSHSA	ATP-dependent RNA helicase DDX42
1524.	QSHSA	Extracellular matrix protein FRAS1 precursor
1525.	QSHSA	Suppressor of IKBKE 1
1526.	SHSAL	Protein tyrosine phosphatase domain-containing protein 1
1527.	SHSAL	Synaptojanin-2
1528.	SHSAL	Zinc finger homeobox protein 3
1529.	SHSAL	DNA excision repair protein ERCC-6-like
1530.	SHSAL	Meiosis inhibitor protein 1
1531.	SHSAL	Xylosyltransferase 1
1532.	SHSAL	Ligand of Numb protein X 2
1533.	SHSAL	DnaJ homolog subfamily C member 5G
1534.	SHSAL	Membrane-spanning 4-domains subfamily A member 14
1535.	SHSAL	X-linked retinitis pigmentosa GTPase regulator-interacting protein 1
1536.	SHSAL	Enhancer of polycomb homolog 1
1537.	SHSAL	Trinucleotide repeat-containing gene 6C protein
1538.	SHSAL	Centrosomal protein of 72 kDa
1539.	SHSAL	E3 ISG15–protein ligase HERC5
1540.	SHSAL	Serine/threonine-protein kinase MRCK beta

(*Continued*)

 © 2023. The Author(s).

Supplementary Table S1 (*Continued*)

1541.	HSALT	Soluble scavenger receptor cysteine-rich domain-containing protein SSC5D precursor
1542.	HSALT	Non-receptor tyrosine-protein kinase TYK2
1543.	HSALT	Protein IL-40 precursor
1544.	HSALT	Zinc finger protein 454
1545.	HSALT	Arginine-glutamic acid dipeptide repeats protein
1546.	SALTA	Fidgetin-like protein 2
1547.	SALTA	Unconventional myosin-Ic
1548.	SALTA	Multidrug resistance-associated protein 6
1549.	SALTA	Dolichyl-diphosphooligosaccharide–protein glycosyltransferase subunit 2 precursor
1550.	SALTA	Perforin-1 precursor
1551.	SALTA	Protein SON
1552.	SALTA	Thrombopoietin receptor precursor
1553.	SALTA	DNA-binding protein inhibitor ID-1
1554.	SALTA	Huntingtin
1555.	SALTA	HMG domain-containing protein 3
1556.	SALTA	Nucleolar and coiled-body phosphoprotein 1
1557.	SALTA	Fidgetin
1558.	SALTA	CRACD-like protein
1559.	SALTA	Zinc transporter ZIP4 precursor
1560.	SALTA	LysM and putative peptidoglycan-binding domain-containing protein 3
1561.	SALTA	Retinitis pigmentosa 1-like 1 protein
1562.	SALTA	Chondroitin sulfate synthase 2
1563.	SALTA	Fez family zinc finger protein 2
1564.	SALTA	Chromodomain-helicase-DNA-binding protein 6
1565.	SALTA	La-related protein 4B
1566.	SALTA	Protein atonal homolog 1
1567.	SALTA	Zinc finger protein with KRAB and SCAN domains 4
1568.	SALTA	Caspase recruitment domain-containing protein 14
1569.	SALTA	Coiled-coil domain-containing protein 90B, mitochondrial precursor
1570.	SALTA	Akirin-1
1571.	SALTA	COP9 signalosome complex subunit 7b
1572.	SALTA	Chromodomain-helicase-DNA-binding protein 8
1573.	SALTA	Zinc finger protein 334
1574.	SALTA	Leucine-rich repeat and fibronectin type-III domain-containing protein 2 precursor
1575.	SALTA	Glucocorticoid modulatory element-binding protein 1
1576.	ALTAL	Uncharacterized protein C2orf81
1577.	ALTAL	Leukocyte immunoglobulin-like receptor subfamily B member 3 precursor
1578.	ALTAL	AP-1 complex subunit gamma-like 2
1579.	ALTAL	Zinc finger and BTB domain-containing protein 7A
1580.	ALTAL	HLA class II histocompatibility antigen, DP beta 1 chain precursor
1581.	ALTAL	Perforin-1 precursor
1582.	ALTAL	Alpha-1D adrenergic receptor
1583.	ALTAL	Sodium- and chloride-dependent creatine transporter 1
1584.	ALTAL	Serine/threonine-protein kinase Nek3

 © 2023. The Author(s).

Supplementary Table S1 (*Continued*)

1585.	ALTAL	Eukaryotic peptide chain release factor subunit 1
1586.	ALTAL	SHC-transforming protein 2
1587.	ALTAL	Di-N-acetyl chitobiose precursor
1588.	ALTAL	Sterol regulatory element-binding protein 2
1589.	ALTAL	E3 ubiquitin-protein ligase TRIP12
1590.	ALTAL	Leucine-rich repeat-containing protein 24 precursor
1591.	ALTAL	E3 ubiquitin-protein ligase UBR4
1592.	ALTAL	Microtubule-associated serine/threonine-protein kinase 2
1593.	ALTAL	Leukocyte immunoglobulin-like receptor subfamily A member 6 precursor
1594.	ALTAL	NLR family CARD domain-containing protein 3
1595.	ALTAL	Probable RNA-binding protein 23
1596.	ALTAL	Serine/threonine-protein kinase 11-interacting protein
1597.	ALTAL	Prenylcysteine oxidase-like precursor
1598.	ALTAL	Thioredoxin domain-containing protein 5 precursor
1599.	ALTAL	Sec1 family domain-containing protein 2
1600.	ALTAL	B-cell linker protein
1601.	ALTAL	Heterogeneous nuclear ribonucleoprotein L-like
1602.	ALTAL	Transcription factor AP-2-beta
1603.	ALTAL	Polyamine deacetylase HDAC10
1604.	ALTAL	MARVEL domain-containing protein 3
1605.	ALTAL	Leucine-rich repeat-containing protein 58
1606.	ALTAL	Small glutamine-rich tetratricopeptide repeat-containing protein beta
1607.	ALTAL	Uncharacterized protein C8orf76
1608.	ALTAL	Telomerase protein component 1
1609.	ALTAL	Alanyl-tRNA editing protein Aarsd1
1610.	ALTAL	Spermatogenesis-associated protein 5-like protein 1
1611.	ALTAL	Caspase recruitment domain-containing protein 14
1612.	ALTAL	Class E basic helix–loop–helix protein 41
1613.	ALTAL	Coiled-coil domain-containing protein 90B, mitochondrial precursor
1614.	ALTAL	Ammonium transporter Rh type B
1615.	ALTAL	Splicing factor, arginine/serine-rich 19
1616.	ALTAL	Nucleolar protein 11
1617.	ALTAL	Actin-related protein 8
1618.	ALTAL	COP9 signalosome complex subunit 7b
1619.	ALTAL	Solute carrier family 52, riboflavin transporter, member 2
1620.	ALTAL	Solute carrier family 52, riboflavin transporter, member 1
1621.	ALTAL	Whirlin
1622.	ALTAL	Ataxin-10
1623.	ALTAL	Exostosin-like 2
1624.	ALTAL	Cip1-interacting zinc finger protein
1625.	ALTAL	SH3 and multiple ankyrin repeat domains protein 1
1626.	ALTAL	LHFPL tetraspan subfamily member 6 protein precursor
1627.	LTALQ	Ankyrin repeat domain-containing protein 63
1628.	LTALQ	Citron Rho-interacting kinase

(*Continued*)

 © 2023. The Author(s).

Supplementary Table S1 (*Continued*)

1629.	LTALQ	DNA repair protein XRCC3
1630.	LTALQ	E3 ubiquitin-protein ligase HERC2
1631.	LTALQ	Phosphate carrier protein, mitochondrial precursor
1632.	LTALQ	Centromere-associated protein E precursor
1633.	LTALQ	Sarcolemmal membrane-associated protein
1634.	LTALQ	BEN domain-containing protein 3
1635.	LTALQ	Acyl-CoA-binding domain-containing protein 5
1636.	LTALQ	E3 ubiquitin-protein ligase LRSAM1
1637.	LTALQ	Retrotransposon Gag-like protein 4
1638.	LTALQ	Organic solute transporter subunit alpha
1639.	LTALQ	Cohesin subunit SA-2
1640.	LTALQ	3-hydroxy-3-methylglutaryl-CoA lyase, cytoplasmic
1641.	LTALQ	Dynein heavy chain 3, axonemal
1642.	LTALQ	Rho guanine nucleotide exchange factor 40
1643.	LTALQ	Importin-4
1644.	LTALQ	B-cell linker protein
1645.	LTALQ	Transcription factor AP-2-beta
1646.	LTALQ	4-hydroxyphenylpyruvate dioxygenase-like protein
1647.	LTALQ	Alpha-N-acetylgalactosaminidase alpha-2,6-sialyltransferase 1
1648.	LTALQ	Kallikrein-14 precursor
1649.	LTALQ	TBC1 domain family member 14
1650.	LTALQ	Stomatin-like protein 1
1651.	TALQT	Protein flightless-1 homolog
1652.	TALQT	WD repeat- and FYVE domain-containing protein 4
1653.	TALQT	Relaxin receptor 2
1654.	TALQT	Helicase with zinc finger domain 2
1655.	TALQT	Centrosomal protein of 44 kDa
1656.	TALQT	Baculoviral IAP repeat-containing protein 6
1657.	TALQT	FACT complex subunit SPT16
1658.	ALQTE	Lysine-specific demethylase 4B
1659.	ALQTE	Proto-oncogene c-Fos
1660.	ALQTE	Coiled-coil domain-containing protein 172
1661.	ALQTE	Mannose-binding protein C precursor
1662.	ALQTE	Alpha-taxilin
1663.	ALQTE	C2 domain-containing protein 3
1664.	ALQTE	Coiled-coil domain-containing protein 40
1665.	ALQTE	Teneurin-4
1666.	ALQTE	Ral GTPase-activating protein subunit beta
1667.	ALQTE	Pleckstrin homology domain-containing family H member 2
1668.	ALQTE	Gamma-taxilin
1669.	ALQTE	Pleckstrin homology domain-containing family H member 1
1670.	LQTEQ	Ankyrin repeat domain-containing protein 34B
1671.	LQTEQ	Tetratricopeptide repeat protein 21A
1672.	LQTEQ	RNA exonuclease 5

 © 2023. The Author(s).

Supplementary Table S1 (*Continued*)

1673.	LQTEQ	E3 ubiquitin-protein ligase RNF170
1674.	LQTEQ	Melanoma inhibitory activity protein 2 precursor
1675.	LQTEQ	cTAGE family member 2
1676.	LQTEQ	A-kinase anchor protein 9
1677.	LQTEQ	Mth938 domain-containing protein
1678.	LQTEQ	Guanylyl cyclase-activating protein 2
1679.	QTEQV	Protein-tyrosine sulfotransferase 1
1680.	QTEQV	WD repeat-containing protein 43
1681.	QTEQV	Dynein heavy chain 5, axonemal
1682.	TEQVQ	Frizzled-6 precursor
1683.	TEQVQ	BTB/POZ domain-containing protein 7
1684.	TEQVQ	IQ motif and SEC7 domain-containing protein 3
1685.	EQVQD	Rho guanine nucleotide exchange factor 35
1686.	EQVQD	Rho guanine nucleotide exchange factor 5
1687.	EQVQD	Ankyrin and armadillo repeat-containing protein
1688.	EQVQD	COX assembly mitochondrial protein homolog
1689.	EQVQD	Dedicator of cytokinesis protein 6
1690.	EQVQD	General transcription factor II-I repeat domain-containing protein 1
1691.	QVQDS	Neuronal PAS domain-containing protein 4
1692.	QVQDS	NACHT, LRR and PYD domains-containing protein 4
1693.	QVQDS	S phase cyclin A-associated protein in the endoplasmic reticulum
1694.	QVQDS	Serine/threonine-protein phosphatase 6 regulatory subunit 1
1695.	VQDSE	Wee1-like protein kinase 2
1696.	VQDSE	Microtubule-associated protein 2
1697.	VQDSE	Elongation factor Tu, mitochondrial precursor
1698.	VQDSE	Dystonin
1699.	VQDSE	SH3 and PX domain-containing protein 2A
1700.	VQDSE	Structure-specific endonuclease subunit SLX4
1701.	VQDSE	Beta/gamma crystallin domain-containing protein 2
1702.	VQDSE	NFX1-type zinc finger-containing protein 1
1703.	QDSEH	Voltage-dependent L-type calcium channel subunit beta-3
1704.	DSEHS	Inactive ubiquitin carboxyl-terminal hydrolase 53
1705.	DSEHS	Reticulophagy regulator 3
1706.	DSEHS	Transcription factor Sp8
1707.	DSEHS	Histone-lysine N-methyltransferase, H3 lysine-36 specific
1708.	DSEHS	UPF0687 protein C20orf27
1709.	DSEHS	SR-related and CTD-associated factor 8
1710.	DSEHS	F-box only protein 7
1711.	SEHSG	DNA polymerase theta
1712.	SEHSG	Poliovirus receptor precursor
1713.	SEHSG	E3 ubiquitin-protein ligase Mdm2
1714.	SEHSG	Protocadherin Fat 4 precursor
1715.	SEHSG	Uncharacterized protein C15orf39
1716.	SEHSG	Thioredoxin domain-containing protein 5 precursor

(*Continued*)

 © 2023. The Author(s).

Supplementary Table S1 (*Continued*)

1717.	SEHSG	Protein prune homolog 2
1718.	SEHSG	Zinc finger protein 778
1719.	SEHSG	Ankyrin repeat and SOCS box protein 6
1720.	SEHSG	A-kinase anchor protein 11
1721.	EHSGK	Protein ANKUB1
1722.	EHSGK	Transcriptional regulator ATRX
1723.	EHSGK	Semaphorin-3A precursor
1724.	EHSGK	A-kinase anchor protein 11
1725.	HSGKM	Transcriptional regulator ATRX
1726.	HSGKM	Dihydropyrimidinase
1727.	SGKMV	SWI/SNF-related matrix-associated actin-dependent regulator of chromatin subfamily A member 5
1728.	SGKMV	TATA-box-binding protein
1729.	SGKMV	Probable global transcription activator SNF2L1
1730.	SGKMV	Transcriptional regulator ATRX
1731.	SGKMV	TATA box-binding protein-like 2
1732.	GKMVA	Cilia- and flagella-associated protein 69
1733.	GKMVA	Myelin P2 protein
1734.	GKMVA	Aminomethyltransferase, mitochondrial precursor
1735.	GKMVA	RNA-binding protein 6
1736.	GKMVA	Nuclear factor of activated T-cells, cytoplasmic 4
1737.	GKMVA	Olfactory receptor 5M9
1738.	GKMVA	Olfactory receptor 5M3
1739.	GKMVA	Olfactory receptor 5M8
1740.	GKMVA	Transient receptor potential cation channel subfamily M member 7
1741.	GKMVA	Talin-1
1742.	KMVAK	Zinc finger protein 723
1743.	KMVAK	Thioredoxin, mitochondrial precursor
1744.	KMVAK	E3 ubiquitin-protein ligase HECTD1
1745.	KMVAK	Protein EFR3 homolog B
1746.	MVAKR	G patch domain-containing protein 2-like
1747.	MVAKR	Pleckstrin homology domain-containing family G member 1
1748.	VAKRQ	E3 ubiquitin-protein ligase parkin
1749.	VAKRQ	CREB3 regulatory factor
1750.	AKRQF	Neuroendocrine convertase 1 precursor
1751.	AKRQF	Protein FAM227B
1752.	AKRQF	Talin-1
1753.	QFRIG	SPRY domain-containing protein 4
1754.	FRIGD	Protein FAM221B
1755.	FRIGD	P2X purinoceptor 6
1756.	FRIGD	C-Jun-amino-terminal kinase-interacting protein 3
1757.	RIGDI	Oocyte-secreted protein 4B precursor
1758.	RIGDI	E3 UFM1-protein ligase 1
1759.	RIGDI	FERM, ARHGEF, and pleckstrin domain-containing protein 2
1760.	RIGDI	NADH-ubiquinone oxidoreductase chain 5

 © 2023. The Author(s).

Supplementary Table S1 (*Continued*)

1761.	RIGDI	FYVE, RhoGEF and PH domain-containing protein 1
1762.	RIGDI	Rho guanine nucleotide exchange factor 28
1763.	RIGDI	F-box/WD repeat-containing protein 5
1764.	RIGDI	FYVE, RhoGEF and PH domain-containing protein 4
1765.	IGDIA	Transcription initiation factor IIB
1766.	GDIAG	Transcription initiation factor IIB
1767.	GDIAG	Dedicator of cytokinesis protein 6
1768.	GDIAG	Ribosome-binding protein 1
1769.	GDIAG	Tight junction protein ZO-2
1770.	DIAGE	52 kDa repressor of the inhibitor of the protein kinase
1771.	DIAGE	Serine/threonine-protein phosphatase 2A regulatory subunit B'' subunit alpha
1772.	DIAGE	Hydrocephalus-inducing protein homolog
1773.	DIAGE	Atlastin-3
1774.	IAGEH	Gamma-glutamylamine cyclotransferase
1775.	IAGEH	Glypican-6 precursor
1776.	AGEHT	Lysine-specific histone demethylase 1A
1777.	AGEHT	Focadhesin
1778.	AGEHT	L-amino-acid oxidase precursor
1779.	GEHTS	Apolipoprotein B-100 precursor
1780.	GEHTS	Ankyrin-3
1781.	GEHTS	Chondroitin sulfate proteoglycan 4 precursor
1782.	GEHTS	Extracellular matrix protein FRAS1 precursor
1783.	GEHTS	Catenin delta-2
1784.	GEHTS	Angiopoietin-related protein 3 precursor
1785.	EHTSF	Beta-crystallin A3
1786.	EHTSF	Sulfotransferase 1A2
1787.	EHTSF	Transmembrane channel-like protein 5
1788.	HTSFD	Sulfotransferase 1C3
1789.	HTSFD	Serine/threonine-protein kinase PAK 6
1790.	HTSFD	Putative trace amine-associated receptor 3
1791.	TSFDK	Uncharacterized protein C3orf84
1792.	TSFDK	Insulin receptor precursor
1793.	TSFDK	Sodium/potassium-transporting ATPase subunit alpha-3
1794.	TSFDK	Dual specificity mitogen-activated protein kinase kinase 4
1795.	TSFDK	Protein FAN
1796.	TSFDK	Protein TALPID3
1797.	TSFDK	Nck-associated protein 1
1798.	SFDKL	Branched-chain amino acid aminotransferase, mitochondrial precursor
1799.	SFDKL	T-box transcription factor TBX1
1800.	SFDKL	T-box transcription factor TBX10
1801.	SFDKL	Regulator of G-protein signaling 20
1802.	SFDKL	T-box transcription factor TBX18
1803.	SFDKL	Replication factor C subunit 4
1804.	SFDKL	Metabotropic glutamate receptor 5 precursor

(*Continued*)

 © 2023. The Author(s).

Supplementary Table S1 (*Continued*)

1805.	SFDKL	Regulator of G-protein signaling 19
1806.	SFDKL	Programmed cell death protein 4
1807.	SFDKL	HEAT repeat-containing protein 5A
1808.	SFDKL	Islet cell autoantigen 1-like protein
1809.	SFDKL	T-box transcription factor TBX15
1810.	SFDKL	Regulator of G-protein signaling 18
1811.	SFDKL	HEAT repeat-containing protein 5B
1812.	FDKLP	AT-rich interactive domain-containing protein 1A
1813.	FDKLP	E3 ubiquitin-protein ligase RNF213
1814.	FDKLP	AT-rich interactive domain-containing protein 1B
1815.	FDKLP	Sodium-dependent phosphate transporter 1
1816.	FDKLP	Osteomodulin precursor
1817.	FDKLP	Probable ATP-dependent RNA helicase DHX35
1818.	FDKLP	F-box/LRR-repeat protein 4
1819.	DKLPE	Tolloid-like protein 1 precursor
1820.	DKLPE	Haptoglobin precursor
1821.	DKLPE	Haptoglobin-related protein
1822.	DKLPE	Transcription factor 7
1823.	DKLPE	Cell surface glycoprotein MUC18 precursor
1824.	DKLPE	Coiled-coil domain-containing protein 92
1825.	DKLPE	Mitogen-activated protein kinase kinase kinase 19
1826.	DKLPE	Proteasome adapter and scaffold protein ECM29
1827.	DKLPE	Uncharacterized protein C1orf94
1828.	DKLPE	Retinoic acid-induced protein 1
1829.	DKLPE	Leucine-rich repeat-containing protein 18
1830.	DKLPE	Probable allantoicase
1831.	DKLPE	Serine/threonine-protein kinase MRCK beta
1832.	KLPEG	Serine/threonine-protein kinase PAK 3
1833.	KLPEG	Integrin alpha-8 precursor
1834.	KLPEG	NGFI-A-binding protein 1
1835.	KLPEG	Mitogen-activated protein kinase kinase kinase 19
1836.	KLPEG	Succinate dehydrogenase assembly factor 4, mitochondrial precursor
1837.	KLPEG	Protein AHNAK2
1838.	KLPEG	Patatin-like phospholipase domain-containing protein 6
1839.	KLPEG	Zinc finger and BTB domain-containing protein 9
1840.	KLPEG	Eukaryotic translation initiation factor 5A-2
1841.	KLPEG	Synaptotagmin-like protein 2
1842.	KLPEG	Kelch-like protein 20
1843.	LPEGG	cTAGE family member 15
1844.	LPEGG	Pecanex-like protein 2
1845.	LPEGG	Agrin precursor
1846.	LPEGG	Pro-opiomelanocortin precursor
1847.	LPEGG	Gelsolin precursor
1848.	LPEGG	Potassium voltage-gated channel subfamily A member 6

 © 2023. The Author(s).

Supplementary Table S1 (*Continued*)

1849.	LPEGG	Transcription factor SOX-9
1850.	LPEGG	NHS-like protein 2
1851.	LPEGG	STE20-related kinase adapter protein alpha
1852.	LPEGG	cTAGE family member 6
1853.	LPEGG	Zinc finger and SCAN domain-containing protein 18
1854.	LPEGG	Flt3-interacting zinc finger protein 1
1855.	LPEGG	Zinc finger protein 407
1856.	LPEGG	Neurexin-2 precursor
1857.	LPEGG	Protein bassoon
1858.	LPEGG	Sal-like protein 2
1859.	LPEGG	Probable RNA-binding protein 19
1860.	LPEGG	Adseverin
1861.	PEGGR	SCO-spondin precursor
1862.	PEGGR	Adenomatous polyposis coli protein 2
1863.	PEGGR	Collagen alpha-2(VI) chain precursor
1864.	PEGGR	Myelin protein P0 precursor
1865.	PEGGR	Transcription factor SOX-9
1866.	PEGGR	Voltage-dependent N-type calcium channel subunit alpha-1B
1867.	PEGGR	RNA-binding protein 20
1868.	PEGGR	Adhesion G protein-coupled receptor A1
1869.	PEGGR	Zinc finger and SCAN domain-containing protein 18
1870.	PEGGR	Conserved oligomeric Golgi complex subunit 8
1871.	PEGGR	Putative protein CLUHP3
1872.	PEGGR	Arf-GAP with GTPase, ANK repeat, and PH domain-containing protein 3
1873.	PEGGR	Homeobox protein Nkx-3.1
1874.	PEGGR	Transcription factor SOX-17
1875.	PEGGR	Calpain-10
1876.	PEGGR	Neurexin-2 precursor
1877.	PEGGR	Zinc finger protein 219
1878.	PEGGR	Arf-GAP with GTPase, ANK repeat, and PH domain-containing protein 1
1879.	EGGRA	Heterogeneous nuclear ribonucleoprotein L
1880.	EGGRA	Ectonucleotide pyrophosphatase/phosphodiesterase family member 1
1881.	EGGRA	DNA mismatch repair protein Msh6
1882.	EGGRA	Oxidoreductase-like domain-containing protein 1
1883.	EGGRA	Extracellular matrix protein FRAS1 precursor
1884.	EGGRA	Serine protease HTRA1 precursor
1885.	EGGRA	Conserved oligomeric Golgi complex subunit 8
1886.	EGGRA	Tribbles homolog 3
1887.	EGGRA	Arf-GAP with GTPase, ANK repeat, and PH domain-containing protein 2
1888.	EGGRA	Zinc finger protein 541
1889.	EGGRA	Jupiter microtubule associated homolog 2
1890.	EGGRA	Calcium-dependent secretion activator 1
1891.	EGGRA	E3 ubiquitin-protein ligase TRIM33
1892.	GGRAT	Putative WAS protein family homolog 3

(*Continued*)

 © 2023. The Author(s).

Supplementary Table S1 (*Continued*)

1893.	GGRAT	Pericentrin
1894.	GGRAT	Metabotropic glutamate receptor 5 precursor
1895.	GGRAT	Nuclear receptor subfamily 0 group B member 1
1896.	GGRAT	WAS protein family homolog 2
1897.	GGRAT	Transmembrane protein 151A
1898.	GGRAT	Zinc finger protein 541
1899.	GGRAT	Chromatin target of PRMT1 protein
1900.	GRATY	Protein FAM246B
1901.	GRATY	Protein FAM246A
1902.	RATYR	Protein phosphatase inhibitor 2 family member C
1903.	RATYR	Phosphoethanolamine/phosphocholine phosphatase
1904.	RATYR	GPI mannosyltransferase 1
1905.	RATYR	Spermatogenesis-associated protein 2
1906.	ATYRG	Proto-oncogene serine/threonine-protein kinase mos
1907.	ATYRG	Uncharacterized aarF domain-containing protein kinase 5
1908.	ATYRG	WSC domain-containing protein 1
1909.	ATYRG	Very large A-kinase anchor protein
1910.	ATYRG	Zinc finger protein 106
1911.	ATYRG	N-alpha-acetyltransferase 60
1912.	ATYRG	Heme-binding protein 1
1913.	TYRGT	Integrin beta-3 precursor
1914.	TYRGT	Zinc finger protein 396
1915.	YRGTA	UHRF1-binding protein 1-like
1916.	YRGTA	Fibrinogen beta chain precursor
1917.	YRGTA	Hepatocyte growth factor-like protein precursor
1918.	YRGTA	Tyrosine-protein kinase transmembrane receptor ROR2 precursor
1919.	YRGTA	Putative macrophage stimulating 1-like protein precursor
1920.	YRGTA	Nucleosome assembly protein 1-like 2
1921.	YRGTA	Tenascin-N precursor
1922.	RGTAF	3-oxo-5-alpha-steroid 4-dehydrogenase 2
1923.	RGTAF	Sodium-dependent phosphate transport protein 2A
1924.	RGTAF	Prolyl endopeptidase FAP
1925.	RGTAF	Dynein assembly factor 4, axonemal
1926.	GTAFG	Alcohol dehydrogenase class-3
1927.	GTAFG	Nuclear pore complex protein Nup98-Nup96 precursor
1928.	GTAFG	Keratin, type II cytoskeletal 72
1929.	GTAFG	Zinc finger protein OZF
1930.	GTAFG	Inactive N-acetylated-alpha-linked acidic dipeptidase-like protein 2
1931.	GTAFG	Olfactory receptor 7G1
1932.	GTAFG	Rap guanine nucleotide exchange factor 4
1933.	GTAFG	Multimerin-2 precursor
1934.	GTAFG	DNA-directed RNA polymerase III subunit RPC2
1935.	GTAFG	Phospholipid-transporting ATPase IA
1936.	TAFGS	Netrin receptor UNC5C precursor

 © 2023. The Author(s).

Supplementary Table S1 (*Continued*)

1937.	TAFGS	Hyaluronan and proteoglycan link protein 1 precursor
1938.	TAFGS	Myosin-10
1939.	TAFGS	Keratin, type II cytoskeletal 72
1940.	TAFGS	TNFAIP3-interacting protein 1
1941.	TAFGS	Putative protein MSS51 homolog, mitochondrial
1942.	TAFGS	Islet cell autoantigen 1-like protein
1943.	AFGSD	Transmembrane protein 182 precursor
1944.	AFGSD	Probable N-acetyltransferase 16
1945.	AFGSD	Serine/threonine-protein kinase Nek9
1946.	FGSDD	Asparagine synthetase [glutamine-hydrolyzing]
1947.	FGSDD	Parathyroid hormone-related protein precursor
1948.	FGSDD	Granulocyte-macrophage colony-stimulating factor receptor subunit alpha precursor
1949.	FGSDD	Elongation factor 1-beta
1950.	FGSDD	Influenza virus NS1A-binding protein
1951.	GSDDA	Homeobox protein cut-like 2
1952.	GSDDA	Guanine nucleotide-binding protein subunit beta-5
1953.	GSDDA	Lysine-specific demethylase PHF2
1954.	GSDDA	Dystrophin
1955.	GSDDA	Guanine nucleotide-binding protein G(I)/G(S)/G(T) subunit beta-3
1956.	GSDDA	Delta-1-pyrroline-5-carboxylate synthase
1957.	GSDDA	Guanine nucleotide-binding protein G(I)/G(S)/G(T) subunit beta-1
1958.	GSDDA	Guanine nucleotide-binding protein G(I)/G(S)/G(T) subunit beta-2
1959.	GSDDA	Cell adhesion molecule 3 precursor
1960.	GSDDA	E3 ubiquitin-protein ligase COP1
1961.	GSDDA	Protein N-terminal asparagine amidohydrolase
1962.	GSDDA	Brother of CDO precursor
1963.	GSDDA	Guanine nucleotide-binding protein subunit beta-4
1964.	GSDDA	TGF-beta-activated kinase 1 and MAP3K7-binding protein 2
1965.	SDDAS	Putative HLA class I histocompatibility antigen, alpha chain H precursor
1966.	SDDAS	Guanine nucleotide-binding protein G(I)/G(S)/G(T) subunit beta-3
1967.	SDDAS	Catenin alpha-1
1968.	SDDAS	E3 ubiquitin-protein ligase NEDD4
1969.	SDDAS	Anoctamin-4
1970.	SDDAS	Uncharacterized protein CXorf66 precursor
1971.	SDDAS	Zinc finger protein 804A
1972.	SDDAS	Serine/threonine-protein kinase DCLK2
1973.	SDDAS	Kin of IRRE-like protein 1 precursor
1974.	SDDAS	Gamma-adducin
1975.	SDDAS	G patch domain-containing protein 8
1976.	DDASG	Calmodulin-regulated spectrin-associated protein 1
1977.	DDASG	BCL-6 corepressor
1978.	DDASG	Ectopic P granules protein 5 homolog
1979.	DDASG	Thrombospondin type-1 domain-containing protein 1 precursor
1980.	DASGK	Tapasin precursor

(*Continued*)

Supplementary Table S1 (*Continued*)

1981.	DASGK	Fatty acid synthase
1982.	DASGK	Zinc finger protein basonuclin-1
1983.	DASGK	FERM and PDZ domain-containing protein 4
1984.	DASGK	Polyadenylate-binding protein 5
1985.	DASGK	LIM domain and actin-binding protein 1
1986.	DASGK	Ras GTPase-activating protein-binding protein 2
1987.	ASGKL	Pleiotropic regulator 1
1988.	ASGKL	Pre-mRNA-splicing factor SLU7
1989.	ASGKL	RNA-binding protein 25
1990.	ASGKL	DNA ligase 4
1991.	ASGKL	Parathyroid hormone/parathyroid hormone-related peptide receptor precursor
1992.	ASGKL	Chromodomain-helicase-DNA-binding protein 4
1993.	ASGKL	Protein ripply2
1994.	ASGKL	Serine protease FAM111B
1995.	ASGKL	Chromodomain-helicase-DNA-binding protein 1-like
1996.	ASGKL	Vacuolar protein sorting-associated protein 8 homolog
1997.	ASGKL	ER membrane protein complex subunit 1 precursor
1998.	ASGKL	Dimethyladenosine transferase 1, mitochondrial precursor
1999.	ASGKL	Ubiquitin carboxyl-terminal hydrolase 7
2000.	ASGKL	Transcription factor 25
2001.	ASGKL	Protein AATF
2002.	ASGKL	Protocadherin beta-8 precursor
2003.	ASGKL	Protocadherin beta-13 precursor
2004.	SGKLT	Kinesin-like protein KIF11
2005.	SGKLT	ADAMTS-like protein 3 precursor
2006.	SGKLT	Dynein heavy chain 14, axonemal
2007.	SGKLT	Otogelin precursor
2008.	SGKLT	Spermidine/spermine N(1)-acetyltransferase-like protein 1
2009.	SGKLT	Dynein heavy chain 10, axonemal
2010.	SGKLT	Adhesion G-protein coupled receptor V1 precursor
2011.	SGKLT	Titin
2012.	SGKLT	Proline-rich protein 11
2013.	SGKLT	Alsin
2014.	SGKLT	Centromere protein O
2015.	SGKLT	Exocyst complex component 1
2016.	GKLTY	Probable ATP-dependent DNA helicase HFM1
2017.	GKLTY	EF-hand calcium-binding domain-containing protein 14
2018.	GKLTY	Myosin-7
2019.	GKLTY	Coiled-coil domain-containing protein 183
2020.	GKLTY	Interleukin-23 receptor precursor
2021.	GKLTY	Low-density lipoprotein receptor-related protein 1B precursor
2022.	GKLTY	Protocadherin gamma-A5 precursor
2023.	KLTYT	Leukotriene A-4 hydrolase
2024.	KLTYT	Myosin-7

 © 2023. The Author(s).

Supplementary Table S1 (*Continued*)

2025.	KLTYT	Serine-protein kinase ATM
2026.	KLTYT	U5 small nuclear ribonucleoprotein 40 kDa protein
2027.	KLTYT	Cilia- and flagella-associated protein 36
2028.	LTYTI	ATP-binding cassette subfamily A member 13
2029.	LTYTI	Protein Aster-A
2030.	TYTID	Protein sidekick-1 precursor
2031.	YTIDF	Sortilin precursor
2032.	TIDFA	Activating signal cointegrator 1
2033.	IDFAA	Reelin precursor
2034.	IDFAA	Zinc transporter 7
2035.	IDFAA	AN1-type zinc finger protein 1
2036.	DFAAK	DENN domain-containing protein 3
2037.	DFAAK	Dermatan-sulfate epimerase-like protein precursor
2038.	DFAAK	Ribonucleoprotein PTB-binding 2
2039.	DFAAK	Sacsin
2040.	DFAAK	Kelch-like protein 13
2041.	FAAKQ	Homogentisate 1,2-dioxygenase
2042.	FAAKQ	7-dehydrocholesterol reductase
2043.	AAKQG	Wolframin
2044.	AAKQG	Nebulin
2045.	AAKQG	DNA polymerase subunit gamma-1
2046.	AAKQG	cAMP-specific 3′,5′-cyclic phosphodiesterase 4C
2047.	AAKQG	Cullin-3
2048.	AAKQG	Ubiquitin carboxyl-terminal hydrolase 31
2049.	AAKQG	Nuclear pore membrane glycoprotein 210 precursor
2050.	AAKQG	Ankyrin repeat and SOCS box protein 15
2051.	AAKQG	Membrane-associated guanylate kinase, WW and PDZ domain-containing protein 1
2052.	AAKQG	Sideroflexin-3
2053.	AAKQG	Vascular non-inflammatory molecule 3 precursor
2054.	AAKQG	Sodium channel protein type 10 subunit alpha
2055.	AKQGH	Growth arrest-specific protein 6 precursor
2056.	AKQGH	Membrane-associated guanylate kinase, WW and PDZ domain-containing protein 1
2057.	AKQGH	Pleckstrin-2
2058.	KQGHG	Histone-lysine N-methyltransferase 2C
2059.	KQGHG	KICSTOR complex protein ITFG2
2060.	KQGHG	Polycomb protein SCMH1
2061.	KQGHG	Serine/threonine-protein kinase PLK2
2062.	QGHGK	Membrane-bound transcription factor site-1 protease precursor
2063.	QGHGK	WASP homolog-associated protein with actin, membranes and microtubules
2064.	GHGKI	Sodium channel subunit beta-2 precursor
2065.	GHGKI	Dystrobrevin alpha
2066.	HGKIE	Mannan-binding lectin serine protease 1 precursor
2067.	HGKIE	Probable ATP-dependent RNA helicase DDX46
2068.	GKIEH	Zinc finger Y-chromosomal protein

(*Continued*)

 © 2023. The Author(s).

Supplementary Table S1 (*Continued*)

2069.	GKIEH	Zinc finger X-chromosomal protein
2070.	GKIEH	DNA damage-binding protein 1
2071.	GKIEH	TBC1 domain family member 2A
2072.	KIEHL	Arf-GAP with dual PH domain-containing protein 1
2073.	KIEHL	TBC1 domain family member 2A
2074.	KIEHL	Rho GTPase-activating protein 35
2075.	IEHLK	RNA helicase aquarius
2076.	IEHLK	Colorectal mutant cancer protein
2077.	IEHLK	DNA ligase 4
2078.	IEHLK	Centromere-associated protein E precursor
2079.	IEHLK	Nucleosome-remodeling factor subunit BPTF
2080.	IEHLK	Centrosomal protein of 128 kDa
2081.	IEHLK	E3 ubiquitin-protein transferase MAEA
2082.	IEHLK	Epididymal-specific lipocalin-9 precursor
2083.	IEHLK	Coiled-coil domain-containing protein 74A
2084.	IEHLK	Spindle and kinetochore-associated protein 1
2085.	IEHLK	Coiled-coil domain-containing protein 74B
2086.	IEHLK	TBC1 domain family member 2A
2087.	IEHLK	Rab3 GTPase-activating protein non-catalytic subunit
2088.	IEHLK	Microtubule-actin cross-linking factor 1, isoforms 1/2/3/5
2089.	IEHLK	Zinc finger protein with KRAB and SCAN domains 5
2090.	EHLKS	Peptide chain release factor 1, mitochondrial precursor
2091.	EHLKS	Colorectal mutant cancer protein
2092.	EHLKS	Myosin-10
2093.	EHLKS	Zinc finger protein 142
2094.	EHLKS	Putative malate dehydrogenase 1B
2095.	EHLKS	Zinc finger protein PLAG1
2096.	EHLKS	Zinc finger protein 584
2097.	EHLKS	Ankyrin repeat and SOCS box protein 10
2098.	EHLKS	F-box/LRR-repeat protein 20
2099.	EHLKS	Structural maintenance of chromosomes protein 6
2100.	EHLKS	Histone acetyltransferase KAT8
2101.	EHLKS	ATP-binding cassette subfamily F member 2
2102.	EHLKS	F-box only protein 40
2103.	EHLKS	Microtubule-actin cross-linking factor 1, isoforms 1/2/3/5
2104.	EHLKS	Hermansky-Pudlak syndrome 5 protein
2105.	HLKSP	B-cell CLL/lymphoma 9 protein
2106.	HLKSP	Neuroblast differentiation-associated protein AHNAK
2107.	HLKSP	Transforming growth factor beta regulator 1
2108.	HLKSP	DBIRD complex subunit ZNF326
2109.	HLKSP	B-cell CLL/lymphoma 9-like protein
2110.	HLKSP	Bestrophin-3
2111.	HLKSP	Whirlin
2112.	HLKSP	Bromodomain adjacent to zinc finger domain protein 2A

 © 2023. The Author(s).

Supplementary Table S1 (*Continued*)

2113.	HLKSP	Sarcosine dehydrogenase, mitochondrial precursor
2114.	LKSPE	ADAM DEC1 precursor
2115.	LKSPE	60S ribosomal protein L4
2116.	LKSPE	Nestin
2117.	LKSPE	Uncharacterized protein C15orf32
2118.	LKSPE	Zinc transporter ZIP12
2119.	LKSPE	DENN domain-containing protein 4C
2120.	LKSPE	Multiple epidermal growth factor-like domains protein 8 precursor
2121.	LKSPE	Sterile alpha motif domain-containing protein 3
2122.	LKSPE	GPI ethanolamine phosphate transferase 3
2123.	LKSPE	Nesprin-2
2124.	LKSPE	Caseinolytic peptidase B protein homolog precursor
2125.	LKSPE	Anoctamin-8
2126.	LKSPE	Ataxin-10
2127.	LKSPE	Paraplegin precursor
2128.	LKSPE	Tyrosine–tRNA ligase, mitochondrial precursor
2129.	KSPEL	T cell receptor alpha variable 12-2 precursor
2130.	KSPEL	Protein FAM227A
2131.	KSPEL	Apolipoprotein A-II precursor
2132.	KSPEL	Arfaptin-2
2133.	KSPEL	Cullin-1
2134.	KSPEL	Zinc finger and BTB domain-containing protein 6
2135.	KSPEL	Protogenin precursor
2136.	KSPEL	DENN domain-containing protein 4C
2137.	KSPEL	Cilia- and flagella-associated protein 47
2138.	KSPEL	Lysophosphatidylserine lipase ABHD12
2139.	KSPEL	Calcium/calmodulin-dependent protein kinase kinase 1
2140.	KSPEL	Retinol dehydrogenase 13
2141.	KSPEL	Uncharacterized protein C9orf43
2142.	KSPEL	Caseinolytic peptidase B protein homolog precursor
2143.	KSPEL	Kinesin-like protein KIF13B
2144.	KSPEL	Ataxin-10
2145.	KSPEL	Zinc finger protein 629
2146.	SPELN	Histone-lysine N-methyltransferase 2A
2147.	SPELN	Shugoshin 2
2148.	SPELN	Putative cystatin-9-like protein CST9LP1 precursor
2149.	SPELN	Protein prune homolog 2
2150.	SPELN	G-protein coupled estrogen receptor 1
2151.	SPELN	Kelch-like protein 31
2152.	SPELN	Exocyst complex component 7
2153.	SPELN	Serine/arginine repetitive matrix protein 2
2154.	SPELN	Complex I intermediate-associated protein 30, mitochondrial precursor
2155.	PELNV	Kelch-like protein 33
2156.	PELNV	Antizyme inhibitor 1

(*Continued*)

Supplementary Table S1 (*Continued*)

2157.	PELNV	Signal-induced proliferation-associated 1-like protein 1
2158.	PELNV	Splicing factor 3B subunit 1
2159.	PELNV	Uncharacterized protein KIAA0754
2160.	PELNV	Steroid 17-alpha-hydroxylase/17,20 lyase
2161.	PELNV	Metabotropic glutamate receptor 7 precursor
2162.	PELNV	Ubiquitin carboxyl-terminal hydrolase 48
2163.	PELNV	Zinc finger protein 710
2164.	ELNVD	Mitogen-activated protein kinase kinase kinase 6
2165.	ELNVD	Serine/threonine-protein phosphatase PP1-beta catalytic subunit
2166.	ELNVD	Highly divergent homeobox
2167.	ELNVD	Cap-specific mRNA (nucleoside-2'-O-)-methyltransferase 1
2168.	ELNVD	Casein kinase I isoform gamma-1
2169.	LNVDL	Tubulin alpha chain-like 3
2170.	LNVDL	Leucine-rich repeats and immunoglobulin-like domains protein 2 precursor
2171.	LNVDL	Tubulin alpha-3C chain
2172.	LNVDL	Tubulin alpha-3D chain
2173.	LNVDL	Tubulin alpha-1B chain
2174.	LNVDL	Tubulin alpha-4A chain
2175.	LNVDL	Protein RRP5 homolog
2176.	LNVDL	Protein GREB1
2177.	LNVDL	Autophagy-related protein 9B
2178.	LNVDL	Tubulin alpha-3E chain
2179.	LNVDL	Tubulin alpha-1A chain
2180.	LNVDL	E3 ubiquitin-protein transferase RMND5B
2181.	LNVDL	Tubulin alpha-1C chain
2182.	LNVDL	Putative tubulin-like protein alpha-4B
2183.	LNVDL	Serine/threonine-protein kinase LATS2
2184.	LNVDL	Tubulin alpha-8 chain
2185.	LNVDL	Ras-related protein Rab-26
2186.	LNVDL	Centrosomal protein of 83 kDa
2187.	NVDLA	Mitochondrial import inner membrane translocase subunit TIM44 precursor
2188.	NVDLA	Nebulin
2189.	NVDLA	Zinc transporter ZIP6 precursor
2190.	NVDLA	Regulation of nuclear pre-mRNA domain-containing protein 2
2191.	NVDLA	HEAT repeat-containing protein 3
2192.	NVDLA	MAGUK p55 subfamily member 5
2193.	NVDLA	Ras-related protein Rab-42
2194.	NVDLA	Rho GTPase-activating protein 35
2195.	NVDLA	Ras-related protein Rab-26
2196.	NVDLA	DmX-like protein 1
2197.	VDLAA	Signal-induced proliferation-associated 1-like protein 3
2198.	VDLAA	Glutamate receptor 2 precursor
2199.	VDLAA	Endogenous retrovirus group S71 member 1 Env polyprotein precursor
2200.	VDLAA	Protein ripply1

 © 2023. The Author(s).

Supplementary Table S1 (*Continued*)

2201.	VDLAA	NAD(P) transhydrogenase, mitochondrial precursor
2202.	VDLAA	Nucleoporin GLE1
2203.	VDLAA	Alpha/beta hydrolase domain-containing protein 17B
2204.	VDLAA	U11/U12 small nuclear ribonucleoprotein 48 kDa protein
2205.	VDLAA	Nicotinate phosphoribosyltransferase
2206.	VDLAA	Probable guanine nucleotide exchange factor MCF2L2
2207.	VDLAA	Neurogenic locus notch homolog protein 4 precursor
2208.	VDLAA	Equilibrative nucleoside transporter 3
2209.	VDLAA	ATP-dependent RNA helicase DDX25
2210.	DLAAS	Putative spermatogenesis-associated protein 31C2
2211.	DLAAS	Multicilin
2212.	DLAAS	Homeobox protein Nkx-2.8
2213.	DLAAS	Signal-induced proliferation-associated 1-like protein 3
2214.	DLAAS	Proto-oncogene tyrosine-protein kinase receptor Ret precursor
2215.	DLAAS	Putative spermatogenesis-associated protein 31C1
2216.	DLAAS	Homeobox protein Nkx-3.2
2217.	DLAAS	Probable E3 ubiquitin-protein ligase HERC1
2218.	DLAAS	Tumor suppressor candidate gene 1 protein
2219.	DLAAS	APC membrane recruitment protein 1
2220.	DLAAS	PDZ domain-containing protein 4
2221.	DLAAS	Calcium homeostasis modulator protein 3
2222.	DLAAS	Retrotransposon Gag-like protein 3
2223.	DLAAS	Putative olfactory receptor 52P1
2224.	DLAAS	Nicotinamide/nicotinic acid mononucleotide adenylyltransferase 3
2225.	DLAAS	Equilibrative nucleoside transporter 3
2226.	DLAAS	Deoxynucleotidyl transferase terminal-interacting protein 1
2227.	DLAAS	StAR-related lipid transfer protein 9
2228.	DLAAS	Dermatan-sulfate epimerase precursor
2229.	DLAAS	Origin recognition complex subunit 6
2230.	DLAAS	SET-binding protein
2231.	LAASD	Sphingosine 1-phosphate receptor 2
2232.	LAASD	Sodium channel protein type 4 subunit alpha
2233.	LAASD	B1 bradykinin receptor
2234.	LAASD	G-protein coupled receptor 15
2235.	LAASD	Polycystin-1 precursor
2236.	LAASD	NK1 transcription factor-related protein 1
2237.	LAASD	Serine/threonine-protein kinase SBK1
2238.	LAASD	Fibrous sheath-interacting protein 2
2239.	LAASD	Potassium channel subfamily T member 1
2240.	LAASD	Myosin-14
2241.	LAASD	Reprimo-like protein
2242.	LAASD	Zinc finger protein GLIS1
2243.	LAASD	Deoxynucleotidyl transferase terminal-interacting protein 1
2244.	LAASD	Lysophosphatidic acid receptor 5

(*Continued*)

 © 2023. The Author(s).

Supplementary Table S1 (*Continued*)

2245.	LAASD	Protocadherin gamma-C4 precursor
2246.	LAASD	Brefeldin A-inhibited guanine nucleotide-exchange protein 1
2247.	LAASD	SET-binding protein
2248.	AASDI	V-set and immunoglobulin domain-containing protein 10-like 2 precursor
2249.	AASDI	14-3-3 protein epsilon
2250.	AASDI	Serine/threonine-protein kinase D1
2251.	AASDI	Lethal(2) giant larvae protein homolog 1
2252.	AASDI	Chromosome alignment-maintaining phosphoprotein 1
2253.	AASDI	Rap guanine nucleotide exchange factor-like 1
2254.	ASDIK	Conserved oligomeric Golgi complex subunit 2
2255.	ASDIK	Cyclin-G2
2256.	ASDIK	Histone-lysine N-methyltransferase KMT5B
2257.	ASDIK	Nebulin-related-anchoring protein
2258.	ASDIK	ATP-binding cassette subfamily F member 1
2259.	SDIKP	Adenylate cyclase type 1
2260.	SDIKP	Apoptosis-stimulating of p53 protein 2
2261.	SDIKP	Rho guanine nucleotide exchange factor 10-like protein
2262.	DIKPD	Apical junction component 1 homolog
2263.	DIKPD	Mitotic checkpoint serine/threonine-protein kinase BUB1
2264.	DIKPD	Serine/threonine-protein kinase LATS1
2265.	DIKPD	Casein kinase I isoform alpha
2266.	DIKPD	Cadherin-8 precursor
2267.	DIKPD	Cadherin-12 precursor
2268.	DIKPD	Myotonin-protein kinase
2269.	DIKPD	Serine/threonine-protein kinase PRP4 homolog
2270.	DIKPD	Apoptosis-stimulating of p53 protein 2
2271.	DIKPD	Serine/threonine-protein kinase 38
2272.	DIKPD	Striated muscle preferentially expressed protein kinase
2273.	DIKPD	Serine/threonine-protein kinase MRCK alpha
2274.	DIKPD	Casein kinase I isoform alpha-like
2275.	DIKPD	Adenosine deaminase domain-containing protein 1
2276.	DIKPD	Protein argonaute-3
2277.	DIKPD	Serine/threonine-protein kinase LATS2
2278.	DIKPD	Serine/threonine-protein kinase 32B
2279.	DIKPD	Protein argonaute-1
2280.	DIKPD	Serine/threonine-protein kinase 38-like
2281.	DIKPD	Serine/threonine-protein kinase MRCK beta
2282.	IKPDK	Guanine nucleotide exchange factor VAV2
2283.	IKPDK	UDP-N-acetylhexosamine pyrophosphorylase
2284.	IKPDK	Protein argonaute-3
2285.	IKPDK	Protein argonaute-1
2286.	KPDKK	Sodium channel protein type 1 subunit alpha
2287.	KPDKK	RPA-related protein RADX
2288.	KPDKK	Sorting nexin-25

 © 2023. The Author(s).

Supplementary Table S1 (*Continued*)

2289.	KPDKK	Ubinuclein-1
2290.	KPDKK	Myosin-15
2291.	PDKKR	Myosin-7B
2292.	PDKKR	Heterogeneous nuclear ribonucleoprotein R
2293.	PDKKR	Ceramide synthase 5
2294.	PDKKR	Probable RNA-binding protein 18
2295.	PDKKR	Fanconi-associated nuclease 1
2296.	DKKRH	Homeobox protein Meis1
2297.	DKKRH	Tuberin
2298.	DKKRH	Heparan-alpha-glucosaminide N-acetyltransferase
2299.	KKRHA	Heparan-alpha-glucosaminide N-acetyltransferase
2300.	KKRHA	SCAN domain-containing protein 3
2301.	KKRHA	Myosin-15
2302.	KRHAV	Receptor-type tyrosine-protein phosphatase zeta precursor
2303.	KRHAV	Tenascin-R precursor
2304.	KRHAV	Homeobox protein Nkx-6.2
2305.	RHAVI	ATP-sensitive inward rectifier potassium channel 8
2306.	RHAVI	Olfactory receptor 14J1
2307.	HAVIS	Inward rectifier potassium channel 4
2308.	HAVIS	G protein-activated inward rectifier potassium channel 2
2309.	HAVIS	G protein-activated inward rectifier potassium channel 1
2310.	HAVIS	Contactin-associated protein 1 precursor
2311.	HAVIS	Nicastrin precursor
2312.	AVISG	Pirin
2313.	AVISG	E3 ubiquitin-protein ligase SIAH2
2314.	AVISG	Solute carrier family 2, facilitated glucose transporter member 10
2315.	AVISG	Collagen alpha-6(IV) chain precursor
2316.	AVISG	SCL-interrupting locus protein
2317.	AVISG	NT-3 growth factor receptor precursor
2318.	AVISG	Serine/threonine-protein kinase MRCK alpha
2319.	AVISG	Transmembrane protein 144
2320.	AVISG	Titin
2321.	AVISG	Apolipoprotein L6
2322.	AVISG	Elongator complex protein 3
2323.	AVISG	Feline leukemia virus subgroup C receptor-related protein 2
2324.	VISGS	E3 ubiquitin-protein ligase Midline-1
2325.	VISGS	Olfactory receptor 2T7
2326.	VISGS	Serine–pyruvate aminotransferase
2327.	VISGS	Anthrax toxin receptor 2 precursor
2328.	VISGS	Protein FAM83E
2329.	VISGS	Olfactory receptor 2T27
2330.	VISGS	Golgi apparatus protein 1 precursor
2331.	VISGS	GREB1-like protein
2332.	VISGS	Protein FAM135A

(*Continued*)

 © 2023. The Author(s).

Supplementary Table S1 (*Continued*)

2333.	VISGS	Angiopoietin-related protein 3 precursor
2334.	ISGSV	Nucleosome-remodeling factor subunit BPTF
2335.	ISGSV	Myotubularin-related protein 1
2336.	ISGSV	Ubiquitin-associated protein 2-like
2337.	ISGSV	Divergent protein kinase domain 1B
2338.	ISGSV	Protein SPT2 homolog
2339.	ISGSV	Uncharacterized protein C15orf39
2340.	ISGSV	RalBP1-associated Eps domain-containing protein 2
2341.	ISGSV	Galectin-12
2342.	ISGSV	Peptide-N(4)-(N-acetyl-beta-glucosaminyl)asparagine amidase
2343.	ISGSV	Zinc finger protein Pegasus
2344.	ISGSV	MAGUK p55 subfamily member 6
2345.	ISGSV	Prenylcysteine oxidase 1 precursor
2346.	ISGSV	A disintegrin and metalloproteinase with thrombospondin motifs 1
2347.	SGSVL	Sialic acid-binding Ig-like lectin 5 precursor
2348.	SGSVL	Glycogenin-2
2349.	SGSVL	Voltage-dependent T-type calcium channel subunit alpha-1G
2350.	SGSVL	Galanin receptor type 2
2351.	SGSVL	DNA polymerase theta
2352.	SGSVL	Mediator of RNA polymerase II transcription subunit 6
2353.	SGSVL	Protocadherin-8 precursor
2354.	SGSVL	Echinoderm microtubule-associated protein-like 2
2355.	SGSVL	Collagen alpha-1(III) chain precursor
2356.	SGSVL	Macrophage colony-stimulating factor 1 precursor
2357.	SGSVL	Serine/threonine-protein kinase A-Raf
2358.	SGSVL	Receptor-type tyrosine-protein phosphatase delta precursor
2359.	SGSVL	Cobalamin binding intrinsic factor precursor
2360.	SGSVL	Guanylate cyclase soluble subunit alpha-2
2361.	SGSVL	Adenylate cyclase type 8
2362.	SGSVL	Transcription factor Dp-2
2363.	SGSVL	GTPase-activating protein and VPS9 domain-containing protein 1
2364.	SGSVL	RNA-binding motif, single-stranded-interacting protein 2
2365.	SGSVL	Tubulin-specific chaperone E
2366.	SGSVL	Adiponectin precursor
2367.	SGSVL	T-box brain protein 1
2368.	SGSVL	Laminin subunit alpha-3 precursor
2369.	SGSVL	1-phosphatidylinositol 4,5-bisphosphate phosphodiesterase eta-1
2370.	SGSVL	Lysophospholipase-like protein 1
2371.	SGSVL	Glutathione hydrolase 6 precursor
2372.	SGSVL	BTB/POZ domain-containing protein KCTD9
2373.	SGSVL	ATP-dependent RNA helicase DDX42
2374.	SGSVL	Cell division cycle protein 20 homolog B
2375.	SGSVL	Homeodomain-interacting protein kinase 1
2376.	SGSVL	Voltage-dependent calcium channel beta subunit-associated regulatory protein

 © 2023. The Author(s).

Supplementary Table S1 (*Continued*)

2377.	SGSVL	Adenylate cyclase type 4
2378.	SGSVL	Rap guanine nucleotide exchange factor 6
2379.	SGSVL	T-complex protein 11 homolog
2380.	SGSVL	Transcription elongation factor, mitochondrial precursor
2381.	SGSVL	Endoplasmic reticulum junction formation protein lunapark
2382.	SGSVL	Ribosomal oxygenase 1
2383.	SGSVL	Anthrax toxin receptor 1 precursor
2384.	SGSVL	Band 4.1-like protein 5
2385.	SGSVL	Semaphorin-3G precursor
2386.	SGSVL	Ubiquitin-associated protein 1
2387.	SGSVL	Histone deacetylase 6
2388.	SGSVL	Polypyrimidine tract-binding protein 2
2389.	SGSVL	Dolichol kinase
2390.	GSVLY	Synaptic vesicular amine transporter
2391.	GSVLY	Zinc finger CCHC domain-containing protein 8
2392.	GSVLY	Contactin-4 precursor
2393.	GSVLY	Progestin and adipoQ receptor family member 4
2394.	GSVLY	Cadherin-23 precursor
2395.	GSVLY	Anoctamin-8
2396.	GSVLY	Cadherin EGF LAG seven-pass G-type receptor 3 precursor
2397.	GSVLY	Low-density lipoprotein receptor-related protein 1B precursor
2398.	GSVLY	TAF6-like RNA polymerase II p300/CBP-associated factor-associated factor 65 kDa subunit 6L
2399.	GSVLY	MAU2 chromatid cohesion factor homolog
2400.	SVLYN	Olfactory receptor 4Q2
2401.	SVLYN	RANBP2-like and GRIP domain-containing protein 1
2402.	SVLYN	RANBP2-like and GRIP domain-containing protein 2
2403.	SVLYN	DNA excision repair protein ERCC-6-like 2
2404.	SVLYN	E3 ubiquitin-protein ligase BRE1A
2405.	SVLYN	Centrosomal protein of 135 kDa
2406.	SVLYN	Solute carrier family 35 member E4
2407.	SVLYN	Transmembrane protease serine 9
2408.	SVLYN	V-set domain-containing T-cell activation inhibitor 1 precursor
2409.	SVLYN	Androglobin
2410.	SVLYN	BPI fold-containing family C protein precursor
2411.	SVLYN	Clusterin-associated protein 1
2412.	SVLYN	Dynein heavy chain 11, axonemal
2413.	VLYNQ	Nidogen-1 precursor
2414.	VLYNQ	MAM and LDL-receptor class A domain-containing protein 1 precursor
2415.	VLYNQ	General transcription factor IIH subunit 4
2416.	VLYNQ	Myozenin-1
2417.	VLYNQ	Signal recognition particle subunit SRP68
2418.	LYNQA	Growth arrest-specific protein 7
2419.	YNQAE	Syntaxin-11
2420.	YNQAE	Protein Wnt-7b precursor

(*Continued*)

 © 2023. The Author(s).

Supplementary Table S1 (*Continued*)

2421.	NQAEK	Prolactin receptor precursor
2422.	NQAEK	Arylsulfatase J precursor
2423.	NQAEK	Glycosyltransferase 1 domain-containing protein 1 precursor
2424.	NQAEK	Protein bicaudal C homolog 1
2425.	QAEKG	Alpha-actinin-4
2426.	QAEKG	Alpha-actinin-2
2427.	QAEKG	Myosin-11
2428.	QAEKG	Peroxisome proliferator-activated receptor delta
2429.	QAEKG	Collagen alpha-1(XVI) chain precursor
2430.	QAEKG	Nesprin-3
2431.	QAEKG	Actin-related protein 8
2432.	AEKGS	Colipase-like protein 1 precursor
2433.	AEKGS	Uroplakin-1a
2434.	AEKGS	Collagen alpha-1(XVI) chain precursor
2435.	AEKGS	Trichohyalin-like protein 1
2436.	AEKGS	Protein enabled homolog
2437.	AEKGS	Protein unc-13 homolog C
2438.	AEKGS	Cilia- and flagella-associated protein 44
2439.	AEKGS	Midasin
2440.	AEKGS	Interleukin-37 precursor
2441.	AEKGS	Potassium voltage-gated channel subfamily D member 2
2442.	AEKGS	Potassium voltage-gated channel subfamily D member 3
2443.	EKGSY	Vitamin K-dependent protein S precursor
2444.	EKGSY	Coagulation factor V precursor
2445.	EKGSY	Ovochymase-2 precursor
2446.	EKGSY	Dynein intermediate chain 3, axonemal
2447.	EKGSY	Protein unc-13 homolog C
2448.	EKGSY	Canalicular multispecific organic anion transporter 1
2449.	EKGSY	Histone-lysine N-methyltransferase ASH1L
2450.	KGSYS	Beta-hexosaminidase subunit beta precursor
2451.	KGSYS	Tyrosine-protein kinase HCK
2452.	KGSYS	Vascular cell adhesion protein 1 precursor
2453.	KGSYS	Myelin transcription factor 1
2454.	KGSYS	Fibrous sheath CABYR-binding protein
2455.	KGSYS	Vitrin precursor
2456.	KGSYS	Canalicular multispecific organic anion transporter 1
2457.	KGSYS	Putative uncharacterized protein C5orf66
2458.	GSYSL	Angiopoietin-related protein 1 precursor
2459.	GSYSL	Beta-hexosaminidase subunit beta precursor
2460.	GSYSL	Tyrosine-protein kinase HCK
2461.	GSYSL	Zinc finger protein 35
2462.	GSYSL	Syndecan-1 precursor
2463.	GSYSL	Vascular cell adhesion protein 1 precursor
2464.	GSYSL	Transcription factor HIVEP2

 © 2023. The Author(s).

Supplementary Table S1 (*Continued*)

2465.	GSYSL	Ras-related protein Rab-40B
2466.	GSYSL	G/T mismatch-specific thymine DNA glycosylase
2467.	GSYSL	Zinc finger protein 474
2468.	GSYSL	Terminal nucleotidyltransferase 4B
2469.	GSYSL	Wiskott-Aldrich syndrome protein family member 1
2470.	GSYSL	Parkin coregulated gene protein
2471.	GSYSL	Specifically androgen-regulated gene protein
2472.	GSYSL	Rho GTPase-activating protein 39
2473.	GSYSL	Putative uncharacterized protein C5orf66
2474.	GSYSL	Src-like-adapter 2
2475.	GSYSL	Angiopoietin-related protein 2 precursor
2476.	SYSLG	Signal transducing adapter molecule 2
2477.	SYSLG	Keratin, type II cytoskeletal 8
2478.	SYSLG	Alpha-2B adrenergic receptor
2479.	SYSLG	Olfactory receptor 8H3
2480.	SYSLG	Olfactory receptor 8H2
2481.	SYSLG	Olfactory receptor 8H1
2482.	SYSLG	Major facilitator superfamily domain-containing protein 8
2483.	SYSLG	S100P-binding protein
2484.	SYSLG	Protocadherin-16 precursor
2485.	SYSLG	Kinesin-like protein KIF13B
2486.	SYSLG	Three-prime repair exonuclease 1
2487.	SYSLG	Protein bassoon
2488.	SYSLG	YTH domain-containing family protein 2
2489.	YSLGI	Relaxin receptor 2
2490.	YSLGI	Zinc finger and BTB domain-containing protein 21
2491.	SLGIF	C-Jun-amino-terminal kinase-interacting protein 4
2492.	SLGIF	Immunoglobulin superfamily member 3 precursor
2493.	SLGIF	Calcitonin receptor precursor
2494.	SLGIF	Sodium/myo-inositol cotransporter
2495.	SLGIF	Calcitonin gene-related peptide type 1 receptor precursor
2496.	SLGIF	Cyclin-Y-like protein 2
2497.	SLGIF	Ovochymase-1 precursor
2498.	SLGIF	Adenosine deaminase domain-containing protein 2
2499.	SLGIF	Kinetochore scaffold 1
2500.	SLGIF	SH3 domain and tetratricopeptide repeat-containing protein 1
2501.	SLGIF	Relaxin receptor 2
2502.	SLGIF	Protein FAM210B, mitochondrial precursor
2503.	SLGIF	Alkaline ceramidase 3
2504.	SLGIF	1-phosphatidylinositol 4,5-bisphosphate phosphodiesterase epsilon-1
2505.	LGIFG	Voltage-dependent T-type calcium channel subunit alpha-1G
2506.	LGIFG	Serine/threonine-protein kinase/endoribonuclease IRE1 precursor
2507.	LGIFG	Calcium-binding mitochondrial carrier protein Aralar1
2508.	LGIFG	Glutathione synthetase

(*Continued*)

 © 2023. The Author(s).

Supplementary Table S1 (*Continued*)

2509.	LGIFG	H(+)/Cl(-) exchange transporter 5
2510.	LGIFG	Sodium/potassium-transporting ATPase subunit beta-1-interacting protein 1
2511.	LGIFG	Carboxylesterase 4A precursor
2512.	LGIFG	Carboxylesterase 5A precursor
2513.	LGIFG	Retinoic acid-induced protein 3
2514.	LGIFG	Sarcoplasmic/endoplasmic reticulum calcium ATPase 3
2515.	LGIFG	Galectin-12
2516.	LGIFG	Sodium-coupled neutral amino acid transporter 3
2517.	LGIFG	Smoothened homolog precursor
2518.	LGIFG	Protocadherin-12 precursor
2519.	LGIFG	Voltage-dependent T-type calcium channel subunit alpha-1I
2520.	LGIFG	1-phosphatidylinositol 4,5-bisphosphate phosphodiesterase epsilon-1
2521.	LGIFG	Carbohydrate sulfotransferase 2
2522.	GIFGG	Keratin, type I cytoskeletal 10
2523.	GIFGG	H(+)/Cl(−) exchange transporter 5
2524.	GIFGG	Glucose-6-phosphate exchanger SLC37A1
2525.	GIFGG	Inositol 1,4,5-trisphosphate receptor type 1
2526.	GIFGG	Ubiquitin carboxyl-terminal hydrolase 10
2527.	GIFGG	WD repeat-containing protein 87
2528.	IFGGQ	WD repeat-containing protein 87
2529.	IFGGQ	Short-chain dehydrogenase/reductase family 42E member 1
2530.	FGGQA	Hsc70-interacting protein
2531.	FGGQA	Putative protein FAM10A5
2532.	FGGQA	Netrin-5 precursor
2533.	FGGQA	TM2 domain-containing protein 2 precursor
2534.	FGGQA	Galactose-3-O-sulfotransferase 2
2535.	GGQAQ	Forkhead box protein D2
2536.	GGQAQ	Carbonic anhydrase 6 precursor
2537.	GGQAQ	Retinoblastoma-like protein 2
2538.	GGQAQ	Zinc finger and BTB domain-containing protein 17
2539.	GGQAQ	Quinone oxidoreductase PIG3
2540.	GGQAQ	Uncharacterized protein KIAA1614
2541.	GGQAQ	Thyroid hormone-inducible hepatic protein
2542.	GGQAQ	CREB-binding protein
2543.	GGQAQ	EEF1A lysine methyltransferase 3
2544.	GGQAQ	Zinc finger protein 469
2545.	GGQAQ	Serine/threonine-protein kinase 32B
2546.	GGQAQ	Cornulin
2547.	GGQAQ	Proline-rich protein 12
2548.	GQAQE	Aryl hydrocarbon receptor nuclear translocator-like protein 1
2549.	GQAQE	Properdin precursor
2550.	GQAQE	Apolipoprotein B receptor
2551.	GQAQE	Ras and Rab interactor 1
2552.	GQAQE	Protein AHNAK2

 © 2023. The Author(s).

Supplementary Table S1 (*Continued*)

2553.	GQAQE	Phospholipid-transporting ATPase ABCA7
2554.	GQAQE	GTPase IMAP family member 8
2555.	GQAQE	Nesprin-2
2556.	GQAQE	Protein-glutamine gamma-glutamyltransferase Z
2557.	GQAQE	Serine/threonine-protein kinase WNK3
2558.	GQAQE	Tyrosine-protein phosphatase non-receptor type 23
2559.	QAQEV	Protein FAM186A
2560.	QAQEV	Triosephosphate isomerase
2561.	QAQEV	Proline-rich protein 9
2562.	QAQEV	Scavenger receptor class A member 3
2563.	QAQEV	Putative 60S ribosomal protein L13a protein RPL13AP3
2564.	QAQEV	Inositol hexakisphosphate and diphosphoinositol-pentakisphosphate kinase
2565.	QAQEV	Leucine-rich repeat-containing protein 52 precursor
2566.	QAQEV	Programmed cell death 6-interacting protein
2567.	QAQEV	Kinesin-like protein KIFC3
2568.	QAQEV	Sialidase-2
2569.	AQEVA	Protein KRBA1
2570.	AQEVA	Coiled-coil domain-containing protein 88B
2571.	AQEVA	Trehalase precursor
2572.	AQEVA	Tetranectin precursor
2573.	AQEVA	Interleukin-6 receptor subunit alpha precursor
2574.	AQEVA	Amiloride-sensitive sodium channel subunit alpha
2575.	AQEVA	N-alpha-acetyltransferase 25, NatB auxiliary subunit
2576.	AQEVA	Protein moonraker
2577.	AQEVA	Uncharacterized protein C1orf167
2578.	AQEVA	pre-rRNA 2’-O-ribose RNA methyltransferase FTSJ3
2579.	AQEVA	UPF0688 protein C1orf174
2580.	AQEVA	Pleckstrin homology domain-containing family G member 4B
2581.	AQEVA	Granulocyte colony-stimulating factor receptor precursor
2582.	AQEVA	CAP-Gly domain-containing linker protein 2
2583.	AQEVA	Unconventional myosin-XV
2584.	AQEVA	Centrosomal protein of 164 kDa
2585.	QEVAG	Transcription intermediary factor 1-alpha
2586.	QEVAG	Zinc finger protein 500
2587.	QEVAG	Disintegrin and metalloproteinase domain-containing protein 11 precursor
2588.	QEVAG	Epidermal growth factor receptor precursor
2589.	QEVAG	Neurogenic locus notch homolog protein 2 precursor
2590.	QEVAG	Zinc finger protein 394
2591.	QEVAG	Protein crumbs homolog 2 precursor
2592.	QEVAG	Zinc finger and SCAN domain-containing protein 29
2593.	QEVAG	UPF0688 protein C1orf174
2594.	QEVAG	MAGUK p55 subfamily member 5
2595.	QEVAG	Neurotrophin receptor-interacting factor homolog
2596.	QEVAG	Resistin precursor

(*Continued*)

 © 2023. The Author(s).

Supplementary Table S1 (*Continued*)

2597.	QEVAG	ATP-binding cassette subfamily F member 3
2598.	QEVAG	Zinc finger protein 446
2599.	QEVAG	Cingulin
2600.	QEVAG	CAP-Gly domain-containing linker protein 2
2601.	EVAGS	TBC1 domain family member 3D
2602.	EVAGS	TBC1 domain family member 3I
2603.	EVAGS	TBC1 domain family member 3E
2604.	EVAGS	TBC1 domain family member 3K
2605.	EVAGS	Coiled-coil domain-containing protein 144A
2606.	EVAGS	TBC1 domain family member 3B
2607.	EVAGS	TBC1 domain family member 3F
2608.	EVAGS	TBC1 domain family member 3L
2609.	EVAGS	Rho guanine nucleotide exchange factor 11
2610.	EVAGS	Trafficking kinesin-binding protein 2
2611.	EVAGS	Pro-epidermal growth factor precursor
2612.	EVAGS	TBC1 domain family member 3H
2613.	EVAGS	Putative uncharacterized protein encoded by LINC00614
2614.	EVAGS	Long-chain-fatty-acid–CoA ligase 1
2615.	EVAGS	Neurogenic locus notch homolog protein 2 precursor
2616.	EVAGS	Glutamine-rich protein 1
2617.	EVAGS	TBC1 domain family member 3G
2618.	EVAGS	TBC1 domain family member 3C
2619.	EVAGS	Putative coiled-coil domain-containing protein 144C
2620.	EVAGS	UPF0688 protein C1orf174
2621.	EVAGS	TBC1 domain family member 3
2622.	EVAGS	G-protein coupled receptor 62
2623.	EVAGS	Resistin precursor
2624.	EVAGS	Matrix metalloproteinase-17 precursor
2625.	VAGSA	Putative 60S ribosomal protein L37a-like protein
2626.	VAGSA	GTPase Era, mitochondrial precursor
2627.	VAGSA	Putative uncharacterized protein FLJ40606
2628.	VAGSA	X-box-binding protein 1
2629.	VAGSA	Cytochrome b-c1 complex subunit 2, mitochondrial precursor
2630.	VAGSA	CAD protein
2631.	VAGSA	Cystatin-D precursor
2632.	VAGSA	Isocitrate dehydrogenase [NAD] subunit gamma, mitochondrial precursor
2633.	VAGSA	Basement membrane-specific heparan sulfate proteoglycan core protein
2634.	VAGSA	Semaphorin-3F precursor
2635.	VAGSA	Nuclear factor of activated T-cells, cytoplasmic 2
2636.	VAGSA	Retinoblastoma-binding protein 5
2637.	VAGSA	Coiled-coil domain-containing protein 142
2638.	VAGSA	Terminal uridylyltransferase 4
2639.	VAGSA	LanC-like protein 3
2640.	VAGSA	Cystin-1

 © 2023. The Author(s).

Supplementary Table S1 (*Continued*)

2641.	VAGSA	MAX gene-associated protein
2642.	VAGSA	Transcription initiation factor TFIID subunit 1-like
2643.	VAGSA	Protein prune homolog 2
2644.	VAGSA	Mitochondrial assembly of ribosomal large subunit protein 1
2645.	VAGSA	Mastermind-like protein 3
2646.	VAGSA	Ubiquitin-like protein 7
2647.	VAGSA	Telomerase Cajal body protein 1
2648.	VAGSA	N-acetyllactosaminide beta-1,3-N-acetylglucosaminyl transferase 4
2649.	VAGSA	Cadherin-23 precursor
2650.	VAGSA	Protein phosphatase 1 regulatory subunit 3E
2651.	VAGSA	Mitochondrial glutamate carrier 1
2652.	VAGSA	Metal transporter CNNM1
2653.	VAGSA	Matrix metalloproteinase-17 precursor
2654.	VAGSA	Transferrin receptor protein 2
2655.	AGSAE	Apical junction component 1 homolog
2656.	AGSAE	Suppressor of cytokine signaling 2
2657.	AGSAE	Histone-lysine N-methyltransferase 2D
2658.	AGSAE	C-Jun-amino-terminal kinase-interacting protein 4
2659.	AGSAE	Musculin
2660.	AGSAE	SLIT and NTRK-like protein 3 precursor
2661.	AGSAE	Formin-like protein 1
2662.	AGSAE	Guanine nucleotide-binding protein G(i) subunit alpha
2663.	AGSAE	Neuromodulin
2664.	AGSAE	X-box-binding protein 1
2665.	AGSAE	Filamin-A
2666.	AGSAE	Cytochrome b-c1 complex subunit 2, mitochondrial precursor
2667.	AGSAE	Diacylglycerol kinase theta
2668.	AGSAE	Liprin-alpha-1
2669.	AGSAE	Neutral amino acid transporter B(0)
2670.	AGSAE	SKI family transcriptional corepressor 2
2671.	AGSAE	Autophagy-related protein 16-1
2672.	AGSAE	Espin-like protein
2673.	AGSAE	Cyclin-Y-like protein 1
2674.	AGSAE	Hemicentin-2 precursor
2675.	AGSAE	Zinc finger and BTB domain-containing protein 45
2676.	AGSAE	Uncharacterized protein FAM167A-AS1
2677.	AGSAE	Histone-lysine N-methyltransferase EHMT1
2678.	AGSAE	Dynein heavy chain 9, axonemal
2679.	AGSAE	ABI gene family member 3
2680.	AGSAE	Mitochondrial import inner membrane translocase subunit Tim22
2681.	GSAEV	Transcription factor SOX-30
2682.	GSAEV	Adenosine deaminase
2683.	GSAEV	Telomere zinc finger-associated protein
2684.	GSAEV	Antigen-presenting glycoprotein CD1d precursor

(*Continued*)

 © 2023. The Author(s).

Supplementary Table S1 (*Continued*)

2685.	GSAEV	Neurexin-1-beta precursor
2686.	GSAEV	Polycystin-1 precursor
2687.	GSAEV	Amyloid-beta A4 precursor protein-binding family A member 1
2688.	GSAEV	Transcription factor E2F4
2689.	GSAEV	MAP7 domain-containing protein 3
2690.	GSAEV	Dyslexia-associated protein KIAA0319-like protein
2691.	GSAEV	Ankyrin repeat domain-containing protein 35
2692.	GSAEV	Myosin-IIIb
2693.	GSAEV	Neurexin-1 precursor
2694.	GSAEV	Potassium voltage-gated channel subfamily H member 3
2695.	SAEVE	Ankyrin repeat domain-containing protein 36A
2696.	SAEVE	Phosphoribosylformylglycinamidine synthase
2697.	SAEVE	Multidrug resistance-associated protein 4
2698.	SAEVE	Synaptojanin-1
2699.	SAEVE	Zinc finger protein 292
2700.	SAEVE	V-type proton ATPase subunit G 2
2701.	SAEVE	Alpha-2-macroglobulin precursor
2702.	SAEVE	Cadherin-2 precursor
2703.	SAEVE	Pregnancy zone protein precursor
2704.	SAEVE	fMet-Leu-Phe receptor
2705.	SAEVE	Heme oxygenase 2
2706.	SAEVE	Amyloid-beta A4 precursor protein-binding family A member 1
2707.	SAEVE	Dystonin
2708.	SAEVE	Bone marrow stromal antigen 2 precursor
2709.	SAEVE	Ankyrin repeat domain-containing protein 36C
2710.	SAEVE	Coiled-coil domain-containing protein 18
2711.	SAEVE	Protein sidekick-1 precursor
2712.	SAEVE	C3 and PZP-like alpha-2-macroglobulin domain-containing protein 8
2713.	SAEVE	Ankyrin repeat domain-containing protein 36B
2714.	SAEVE	Histone acetyltransferase KAT6B
2715.	SAEVE	Selenocysteine insertion sequence-binding protein 2-like
2716.	SAEVE	Autophagy-related protein 2 homolog B
2717.	SAEVE	Unconventional myosin-XVB
2718.	SAEVE	Coiled-coil domain-containing protein 3 precursor
2719.	SAEVE	Sacsin
2720.	SAEVE	Solute carrier family 12 member 7
2721.	AEVET	Dihydrolipoyllysine-residue acetyltransferase component of pyruvate dehydrogenase complex, mitochondrial precursor
2722.	AEVET	Heme oxygenase 2
2723.	AEVET	Centromere protein F precursor
2724.	AEVET	Gasdermin-D
2725.	AEVET	Armadillo repeat-containing X-linked protein 4
2726.	AEVET	Probable ribonuclease ZC3H12B
2727.	AEVET	Exocyst complex component 3-like protein
2728.	AEVET	Coiled-coil alpha-helical rod protein 1

 © 2023. The Author(s).

Supplementary Table S1 (*Continued*)

2729.	AEVET	Sushi, nidogen, and EGF-like domain-containing protein 1 precursor
2730.	AEVET	THO complex subunit 3
2731.	AEVET	Exocyst complex component 2
2732.	AEVET	Transcription factor IIIB 50 kDa subunit
2733.	AEVET	Nuclear receptor-binding protein
2734.	AEVET	Sialidase-2
2735.	EVETA	Phosphatidylinositol 4-phosphate 5-kinase type-1 gamma
2736.	EVETA	Spliceosome RNA helicase DDX39B
2737.	EVETA	Protein ZGRF1
2738.	EVETA	Androglobin
2739.	EVETA	Probable RNA-binding protein EIF1AD
2740.	EVETA	Cysteine-rich secretory protein LCCL domain-containing 2 precursor
2741.	EVETA	RING finger protein 39
2742.	VETAN	Placenta growth factor precursor
2743.	VETAN	A-kinase anchor protein 9
2744.	VETAN	McKusick-Kaufman/Bardet-Biedl syndromes putative chaperonin
2745.	VETAN	Phospholipid-transporting ATPase IB
2746.	ETANG	Probable ubiquitin carboxyl-terminal hydrolase FAF-Y
2747.	ETANG	TRAF-type zinc finger domain-containing protein 1
2748.	ETANG	Putative uncharacterized protein FLJ45684
2749.	ETANG	Capping protein inhibiting regulator of actin dynamics
2750.	ETANG	Draxin precursor
2751.	ETANG	Probable carboxypeptidase X1 precursor
2752.	ETANG	Potassium channel subfamily K member 12
2753.	TANGI	Pre-mRNA-processing-splicing factor 8
2754.	TANGI	Capping protein inhibiting regulator of actin dynamics
2755.	TANGI	Dentin sialophosphoprotein precursor
2756.	ANGIR	E3 ubiquitin-protein ligase MARCHF6
2757.	ANGIR	Pyruvate dehydrogenase phosphatase regulatory subunit, mitochondrial
2758.	NGIRH	Matrix metalloproteinase-9 precursor
2759.	NGIRH	Succinate–CoA ligase [ADP/GDP-forming] subunit alpha, mitochondrial
2760.	NGIRH	ATP-binding cassette subfamily A member 13
2761.	NGIRH	Succinate dehydrogenase cytochrome b560 subunit, mitochondrial
2762.	GIRHI	Splicing factor 3B subunit 3
2763.	GIRHI	Acid-sensing ion channel 2
2764.	GIRHI	NADP-dependent malic enzyme, mitochondrial precursor
2765.	RHIGL	Lysosomal-trafficking regulator
2766.	RHIGL	Harmonin
2767.	HIGLA	Ran-binding protein 6
2768.	HIGLA	Dynein heavy chain 14, axonemal
2769.	HIGLA	Pumilio homolog 1
2770.	HIGLA	Olfactory receptor 52B4
2771.	IGLAA	Integrin beta-3 precursor
2772.	IGLAA	Adenosylhomocysteinase

(*Continued*)

 © 2023. The Author(s).

Supplementary Table S1 (*Continued*)

2773.	IGLAA	Protein VAC14 homolog
2774.	IGLAA	TATA box-binding protein-associated factor RNA polymerase I subunit C
2775.	IGLAA	Coiled-coil domain-containing protein 142
2776.	IGLAA	Magnesium transporter NIPA1
2777.	IGLAA	Integrator complex subunit 1
2778.	IGLAA	C-type lectin domain family 1 member A
2779.	IGLAA	4-hydroxybenzoate polyprenyltransferase, mitochondrial precursor
2780.	IGLAA	Adenylate cyclase type 10
2781.	IGLAA	Cytochrome b-245 chaperone 1
2782.	IGLAA	Transmembrane protein 260
2783.	IGLAA	HMG domain-containing protein 4
2784.	GLAAK	Zinc finger SWIM domain-containing protein 8
2785.	GLAAK	Insulin-induced gene 1 protein
2786.	GLAAK	Microtubule-associated serine/threonine-protein kinase 3
2787.	GLAAK	Deoxyuridine 5′-triphosphate nucleotidohydrolase, mitochondrial precursor
2788.	GLAAK	N-alpha-acetyltransferase 20
2789.	GLAAK	Heat shock 70 kDa protein 14
2790.	GLAAK	Germ cell-specific gene 1 protein
2791.	GLAAK	Ankyrin repeat domain-containing protein SOWAHA precursor
2792.	GLAAK	Glutamate-rich protein 1
2793.	GLAAK	Protein FAM9A
2794.	GLAAK	Serine/threonine-protein kinase PAK 6
2795.	GLAAK	Serine palmitoyltransferase 3
2796.	GLAAK	Core histone macro-H2A.2
2797.	LAAKQ	Nucleolar pre-ribosomal-associated protein 1
2798.	LAAKQ	Wolframin
2799.	LAAKQ	Nebulin
2800.	LAAKQ	40S ribosomal protein S4, X isoform
2801.	LAAKQ	Heat shock 70 kDa protein 14
2802.	LAAKQ	Spliceosome-associated protein CWC27 homolog
2803.	LAAKQ	Transcription factor AP-2-epsilon
2804.	LAAKQ	Probable guanine nucleotide exchange factor MCF2L2
2805.	LAAKQ	40S ribosomal protein S4, Y isoform 2
2806.	LAAKQ	Vascular non-inflammatory molecule 3 precursor
2807.	LAAKQ	Protein inturned
2808.	LAAKQ	Chromatin-remodeling ATPase INO80
2809.	LAAKQ	Histone deacetylase 5

 © 2023. The Author(s).
